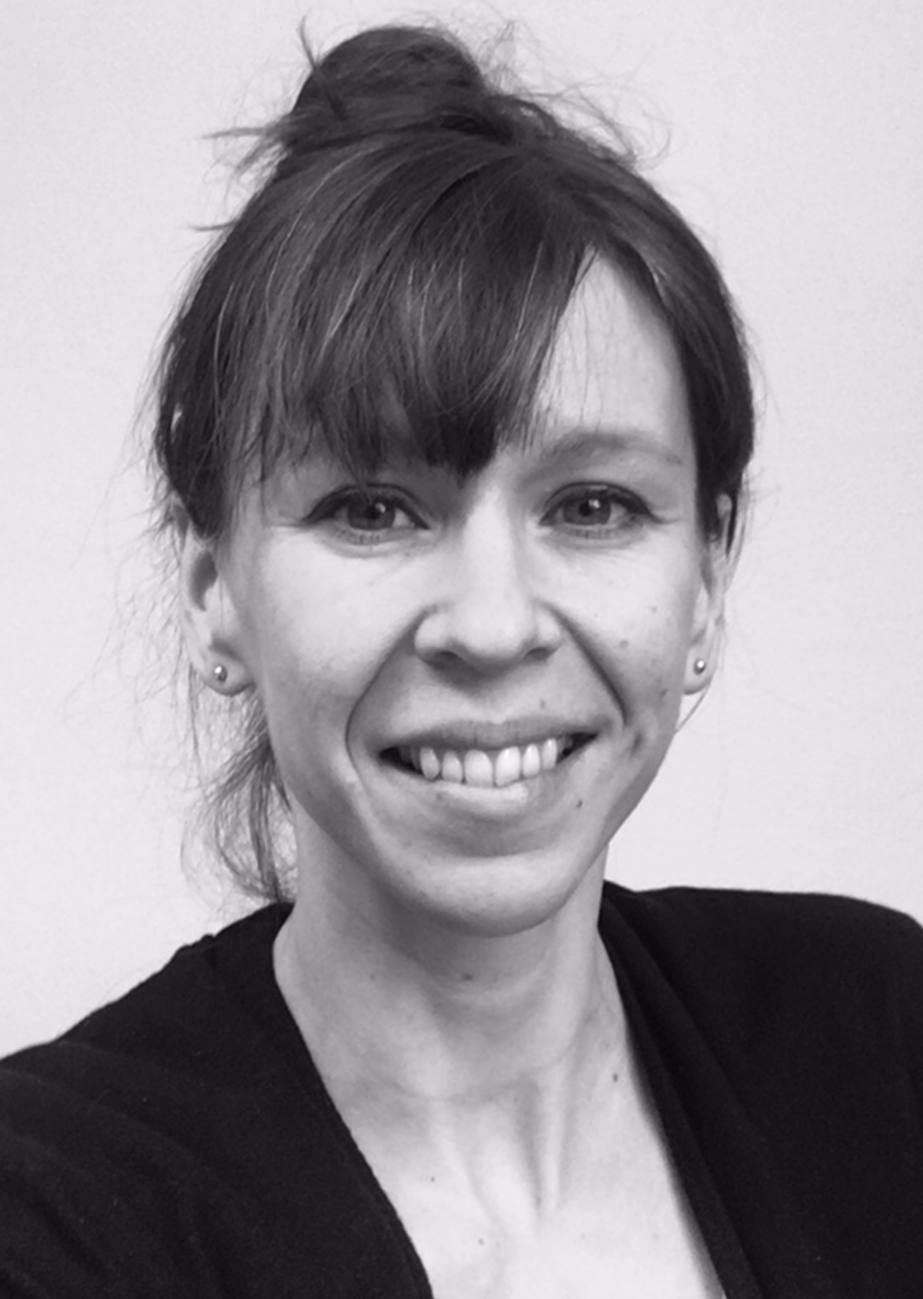# Electrosynthetic C−O Bond Activation in Alcohols and Alcohol Derivatives

**DOI:** 10.1002/anie.202211952

**Published:** 2022-12-05

**Authors:** Piret Villo, Andrey Shatskiy, Markus D. Kärkäs, Helena Lundberg

**Affiliations:** ^1^ Department of Chemistry KTH Royal Institute of Technology SE-100 44 Stockholm Sweden

**Keywords:** Alcohol, C−O Bond Activation, Cathodic Reduction, Deoxygenative, Electrosynthesis

## Abstract

Alcohols and their derivatives are ubiquitous and versatile motifs in organic synthesis. Deoxygenative transformations of these compounds are often challenging due to the thermodynamic penalty associated with the cleavage of the C−O bond. However, electrochemically driven redox events have been shown to facilitate the C−O bond cleavage in alcohols and their derivatives either through direct electron transfer or through the use of electron transfer mediators and electroactive catalysts. Herein, a comprehensive overview of preparative electrochemically mediated protocols for C−O bond activation and functionalization is detailed, including direct and indirect electrosynthetic methods, as well as photoelectrochemical strategies.

## Graphical abstract

Deoxygenative transformations of alcohols and their derivatives are often challenging due to the thermodynamic penalty associated with the cleavage of the C−O bond. This Review includes preparative protocols and their mechanistic aspects for the activation and functionalization of C−O bonds, detailing direct and indirect electrosynthetic methods, as well as photoelectrochemical strategies.

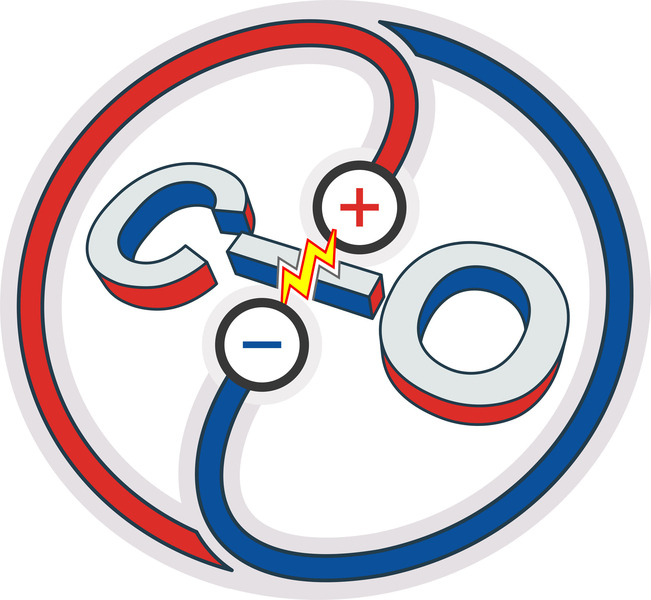

## 1 Introduction

**Figure 1:**
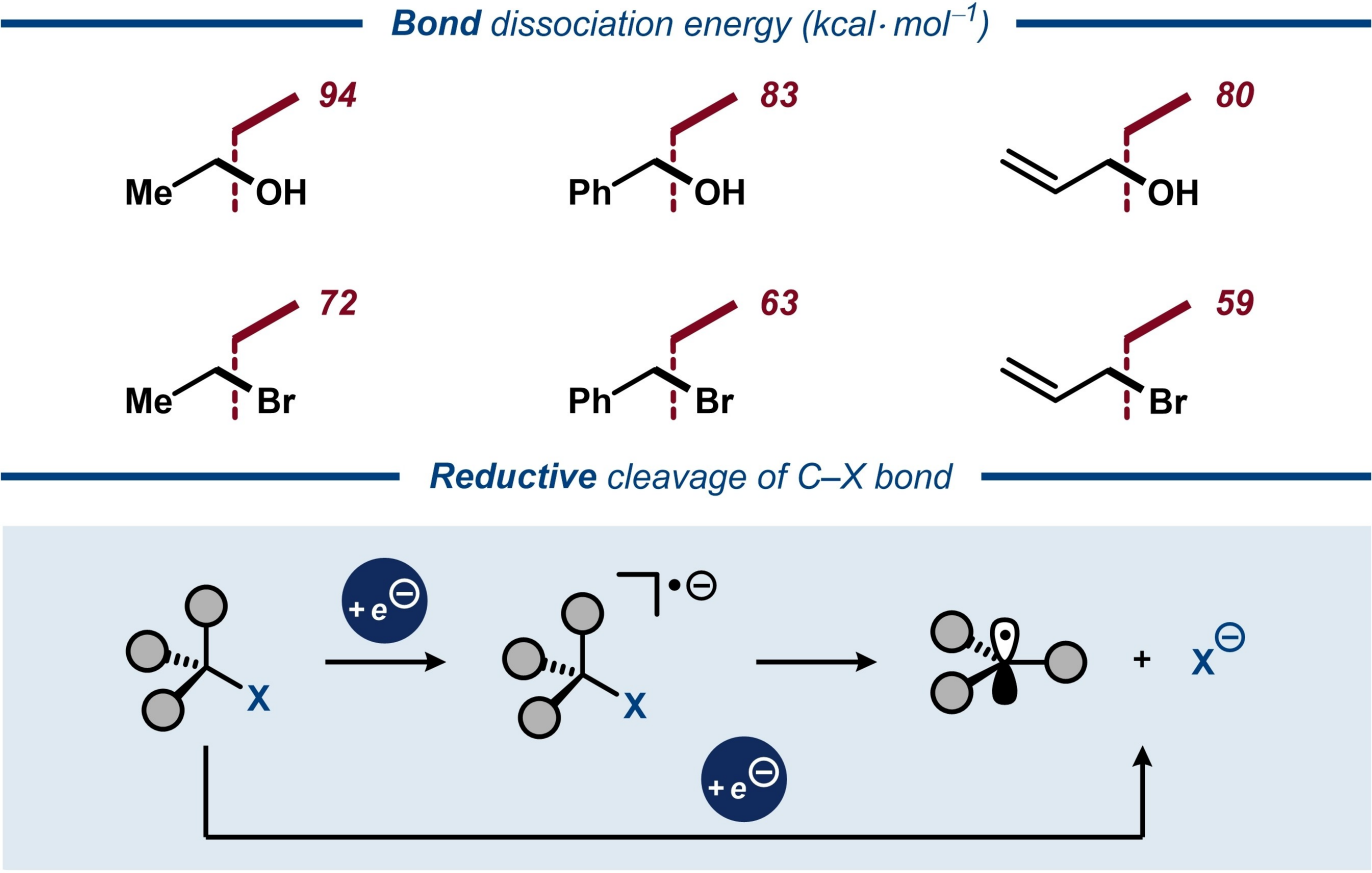
Top: Bond dissociation energies for aliphatic alcohols and the corresponding bromides. Bottom: Reductive C−X bond cleavage through stepwise and concerted dissociative electron transfer.

Alcohols constitute a highly prevalent and versatile class of substrates in organic chemistry. The hydroxy group is found in everything from natural products and biopolymers to pharmaceutically active compounds and serves as a ubiquitous synthetic handle for a multitude of transformations. Despite the broad reactivity profile of alcohols, selective cleavage of the C−OH bond through either heterolytic or homolytic pathways presents an eminent synthetic challenge due to the highly endothermic nature of such processes. The higher thermodynamic penalty for the cleavage of the C−O bond relative to other polarized σ‐bonds, such as C−Br, arises almost exclusively from the higher energy of the resulting O‐centered radical or anion fragments. This manifests in consistently higher (by 20–22 kcal mol^−1^) bond dissociation energies (BDEs) for C−O bonds relative to C−Br bonds in a series of related compounds (Figure 1, top).^[1]^

A handful of catalytic strategies have been developed in order to overcome the intrinsic inertness of the C−OH bond, including redox‐based protocols^[2]^ and various dehydration strategies mediated by Brønsted or Lewis acid catalysts.^[3]^ Activation of C−OH bonds using the latter approach is frequently limited to alcohols with adjacent π‐conjugated systems, which promotes cleavage of the C−OH bond by stabilizing the resulting carbocation intermediates.^[1]^ Such π‐activation also enables deoxygenative cross‐coupling of alcohols and their derivatives by transition metal catalysis in Tsuji–Trost‐type allylation reactions, where C−O bond cleavage is achieved upon oxidative addition to a low‐valent transition metal catalyst.^[4]^ Despite these developments, general catalytic methods for direct C−O bond activation in aliphatic alcohols are scarce,^[5]^ and the majority of protocols rely on stoichiometric derivatization of the alcohol functionality to enable subsequent reduction, nucleophilic substitution or transition metal‐catalyzed cross‐coupling reactions.

**Figure 2:**
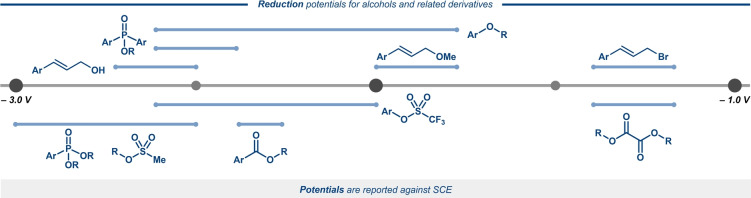
Approximate reduction potentials of alcohols and alcohol derivatives.

The chemistry of carbon‐centered radicals (C‐radicals) has been rapidly expanding during the last decade. A common strategy for generation of such species is one‐electron reduction of alkyl iodides and bromides. Here, the bond cleavage is onset by a dissociative electron transfer, which proceeds either via a stepwise formation of an anion‐radical followed by its decomposition into a C‐radical and the halide anion, or via the corresponding concerted mechanism (Figure 1, bottom).^[6]^ These approaches have been widely capitalized on in recent years, in particular for cross‐electrophile coupling reactions.^[7]^ In comparison with C−Br and C−I bonds, direct reduction of C−OH bonds requires significantly more negative potentials even for π‐activated alcohols.[^8^, ^9^] Hence, classical strategies for C−O bond activation, such as the Barton–McCombie reaction, require pre‐derivatization of the alcohol to facilitate bond scission.^[10]^ Figure 2 provides an overview of reduction potentials for various alcohol derivatives.[^8^, ^9^, ^11^, ^12^, ^13^, ^14^, ^15^, ^16^, ^17^, ^18^]

Electrosynthesis constitutes an emerging synthetic strategy for harnessing the unique reactivity of radical intermediates. In electrosynthetic cells, the electrical bias either provides driving force for the reaction or supplies energy for surmounting activation barriers in the key steps of the desired transformation.^[19]^ Currently, electrosynthesis experiences a wide revival, to a significant extent driven by its application for the synthesis of complex organic molecules.^[20]^ The majority of electrosynthetic protocols rely on direct electrolysis, where electron transfer to or from the electroactive substrate occurs at the electrode surface.^[21]^ In contrast, indirect electrolysis utilizes a redox‐active species for onsetting the desired reactivity. This category includes mediated electrosynthesis that utilizes redox shuttles to transfer charge from the electrode to a substrate or reactive intermediate via outer‐sphere electron transfer, circumventing slow heterogeneous electron transfer kinetics and increasing the selectivity of the reaction.^[22]^ An alternative class of indirect electrosynthetic protocols, sometimes denoted as “metallaelectrocatalysis”, utilizes the applied bias for (re)generation of the active oxidation state of a transition metal catalyst or changing oxidation state of the catalyst within the catalytic cycle.^[23]^ Despite the great potential of electrosynthesis for selective and sustainable transformations, this approach remains surprisingly underexplored in the context of C−O bond activation and functionalization. With the aim of spurring further interest and innovation in this field, this Review provides an overview of synthetically relevant electrochemical strategies for deoxygenative transformations of alcohols and alcohol derivatives. The Review is divided with respect to the class of the electroactive compound that undergoes the C−O bond cleavage and encompasses both direct and indirect electrosynthetic methods. To facilitate comparisons between systems, all potentials are reported versus saturated calomel electrode (SCE). In cases where other reference electrodes were used in the original reports, the herein reported values were calculated based on the previously reported conversion constants.^[24]^

## 2 Electrochemical C−O Bond Activation in Unfunctionalized Alcohols

**Figure 3:**
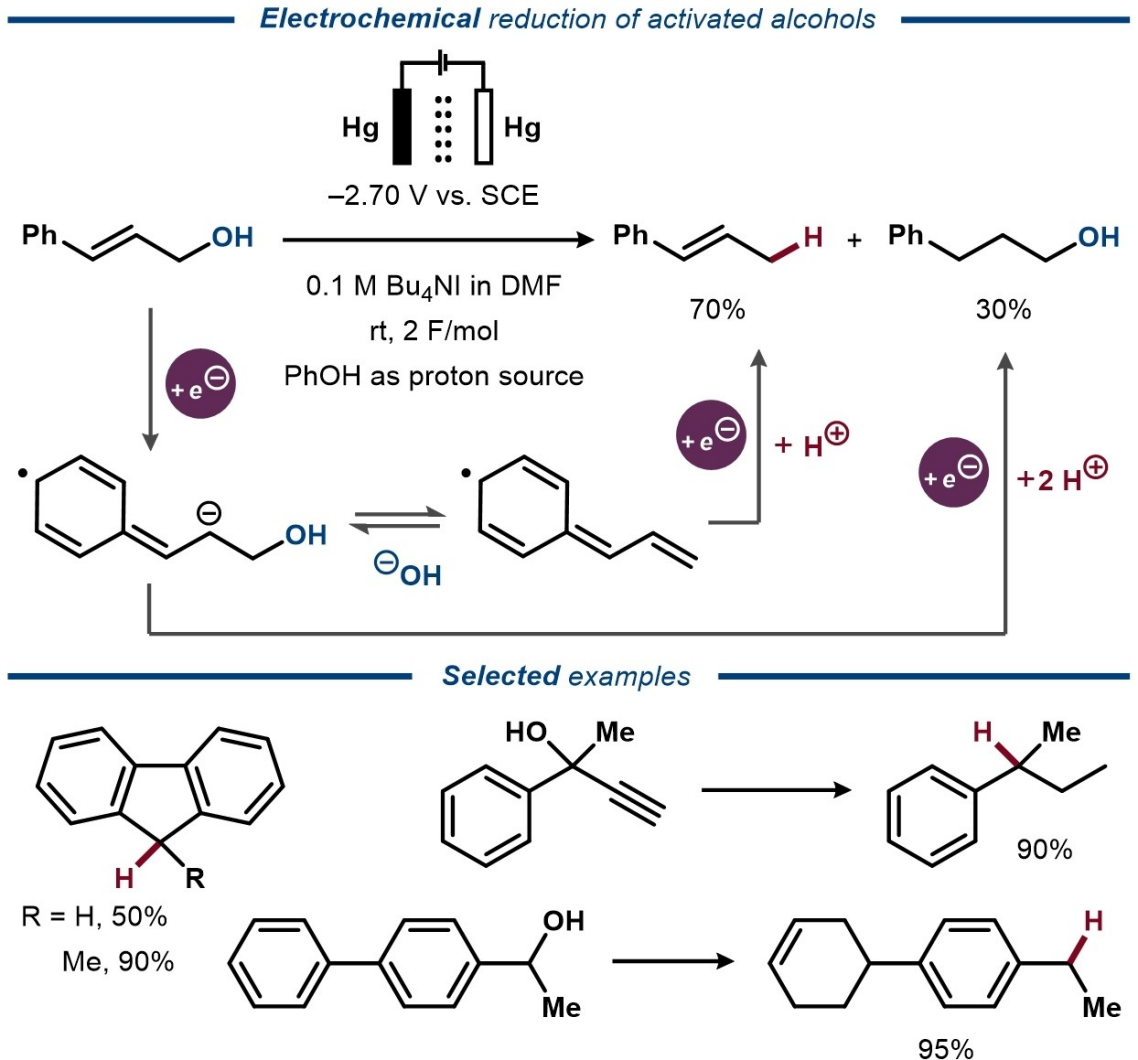
Electroreductive deoxygenation of benzylic and allylic alcohols.

The high BDE of the C−O bond and the poor leaving group ability of the OH‐group is reflected in the limited number of protocols for direct electrochemical reduction of alcohols to the corresponding alkanes. Of those reported, all refer to alcohols with adjacent π‐conjugated systems that stabilize high energy intermediates, similar to what is commonly observed for related two‐electron reactions.^[25]^ One of the earliest protocols for direct electroreduction of a C−OH bond was reported by Given and Peover in 1959 for the reduction of xanthydrol to xanthene with phenol as proton source.^[26]^ Similarly, Lund and co‐workers demonstrated the electrochemical reduction of an extensive scope of π‐activated alcohols under similar electroreductive conditions in the 1970s (Figure 3).[^8^, ^27^] For some of the unsaturated substrates, alkenes or alkynes were reduced prior to C−O bond cleavage, furnishing saturated alcohols alongside the deoxygenation products, while pinacols were shown to undergo either C−C and/or C−O bond cleavage, depending on the used additive. Electroreduction of a series of unsaturated alcohols was further demonstrated by Lund and Lund in 1984.^[28]^ The reaction was proposed to proceed via protonation of an in situ formed alkylmercuric iodide intermediate by the mercury pool cathode in the presence of NaI. A similar approach was applied for electroreduction of biomass‐based furfural derivatives to hydrocarbons in a two‐phase system.^[29]^

In the 1970s and 1980s, Horányi and co‐workers studied the formation of saturated hydrocarbons by cathodic reduction of allylic and propargylic alcohols.^[30]^ For allylic alcohols, C−OH cleavage was proposed to occur first, followed by reduction of the C−C double bond, whereas reduction of the triple bond in propargylic alcohols was shown to precede C−OH bond cleavage. Saturated aliphatic alcohols did not furnish deoxygenation products. Indirect electrochemical deoxygenation of 2‐propanol and 2‐butanol was also reported.^[31]^ Here, secondary alcohols were proposed to undergo anodic oxidation, followed by cathodic reduction of the formed ketone to the corresponding hydrocarbon.

**Figure 4:**
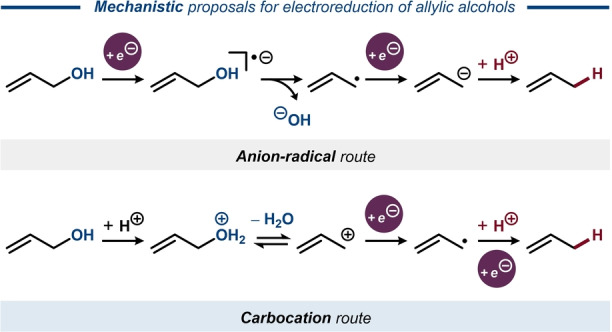
Mechanistic proposals for electroreduction of allyl alcohol to propene.

Electroreduction of allyl alcohol to propene and propane in acidic media has been reported by several groups.[^30^, ^32^, ^33^] The overall transformation is a two‐electron reaction that includes C−OH bond cleavage, formation of propene, and subsequent reduction of the latter to propane. However, the mechanism has been a subject of debate (Figure 4). Horányi and Torkos suggested that formation of an oxonium ion and its conversion to the corresponding allyl cation takes place prior to electron transfer that furnishes an allyl radical.^[34]^ In contrast, Liu and co‐workers suggested that one‐electron reduction occurs first, giving rise to an anion‐radical that dissociates into an allyl radical and a hydroxide ion.^[32]^ Subsequently, Shukun and co‐workers deemed the formation of the initial anion‐radical unlikely in acidic media, supporting the carbocationic route.^[35]^

**Figure 5:**
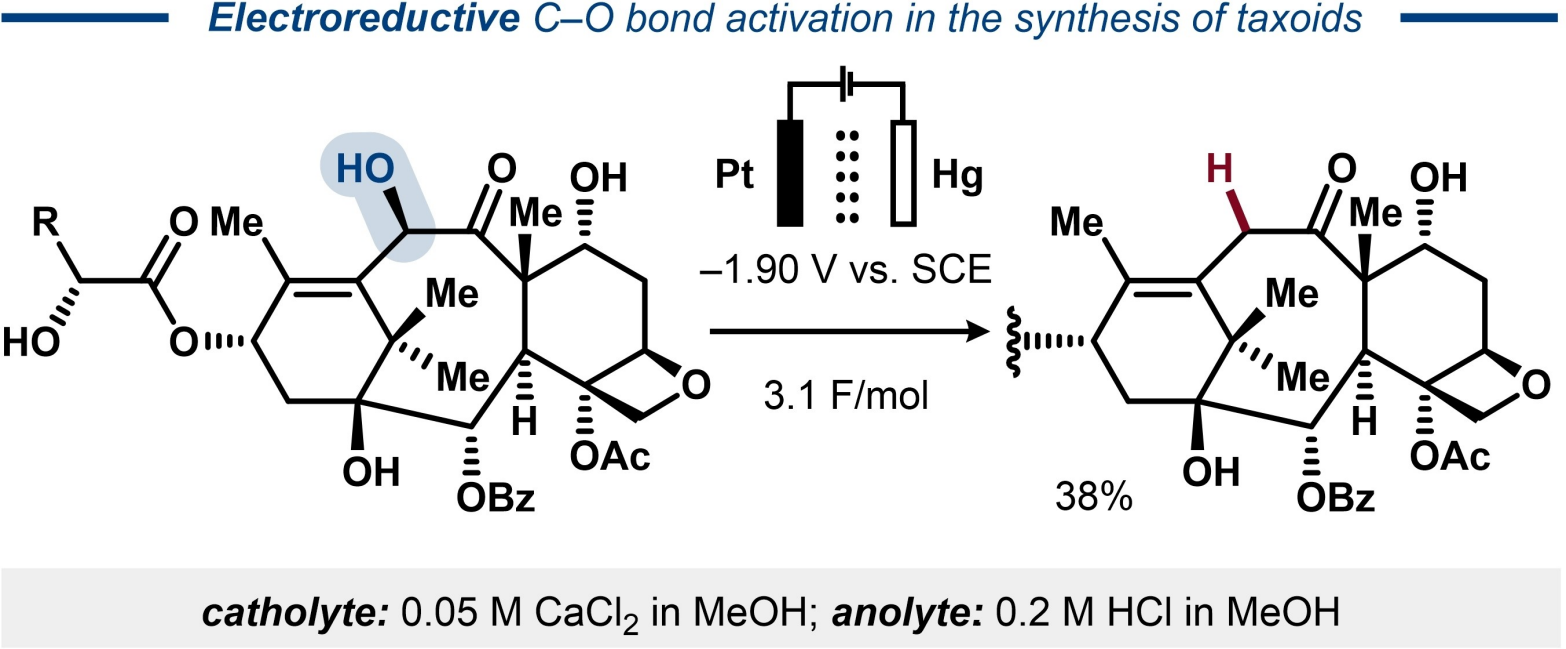
Electroreductive deoxygenation in the synthesis of taxoids.

In 1994, direct electroreduction of an allylic alcohol functionality in complex natural products was demonstrated by Commerçon in the synthesis of taxoids.[^36^, ^37^] The deoxygenation was shown to be highly selective, leaving intact a variety of functional groups, such as ketone, ether, aliphatic alcohols and esters (Figure 5).

## 3 Electrochemical C−O Bond Activation in Ethers

Similar to electrochemical C−OH bond activation in alcohols, adjacent π‐conjugated systems facilitate deoxygenative C−O bond cleavage in ethers.^[38]^ Direct electroreduction of ethers typically proceeds via initial formation of an anion‐radical, followed by its fragmentation to a C‐radical and an alkoxide anion.^[39]^ The chemoselectivity of the fragmentation step is determined by the stability of the formed free‐radical species. In the presence of an adjacent π‐conjugated system, the C−O bond cleavage typically proceeds to form a π‐stabilized radical.[^40^, ^41^] This selectivity has been widely used for reductive removal of ether protecting groups.[^42^, ^43^, ^44^, ^45^] Early examples include a potentiostatic protocol by Utley and co‐workers from 1973 for reductive cleavage of C−O bonds in electron‐deficient benzylic methyl ethers to form the corresponding toluene products.^[17]^ In addition to the methyl group, acetates and benzylic fluorides were removed under the same conditions. Similarly, electrochemical hydrogenation of aryl alkyl ethers to phenols and cyclohexanols[^46^, ^47^, ^48^] as well as of α‐keto ethers[^49^, ^50^] have been reported for lignin model compounds.^[51]^ Electroreduction of diaryl ethers has also been disclosed, furnishing phenols, cyclohexanols or partially saturated ether products.[^52^, ^53^, ^54^, ^55^] In the 1980s, Bartak and co‐workers studied the electrochemical behavior of cyano‐substituted anisoles and diaryl ethers that were found to form a mixture of products, including deoxygenated biaryls, under electroreductive conditions.[^56^, ^57^, ^58^, ^59^] Analogous dimerization of cation‐radical species formed from benzylic methoxy ethers under electrooxidative conditions has also been reported,^[60]^ as well as electrochemically induced C−O bond cleavage in acyclic ethers.[^18^, ^61^, ^62^, ^63^, ^64^]

**Figure 6:**
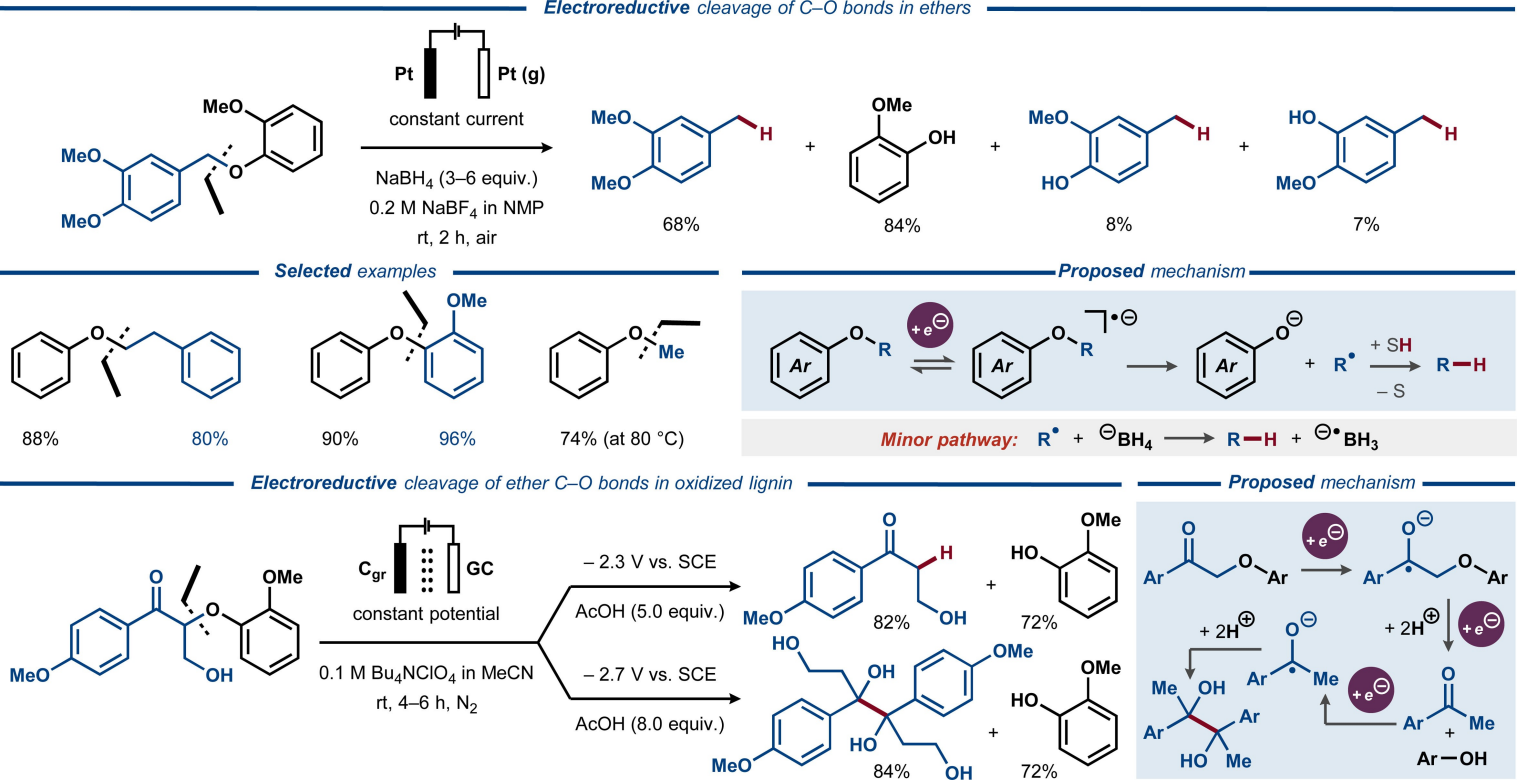
Electrochemical C−O bond cleavage in phenolic ethers. Top: Electroreductive cleavage of C−O bonds in diaryl and alkyl aryl ethers in the presence of NaBH_4_ (Pt(g) = platinum minigrid). Bottom: Electroreductive cleavage of oxidized lignin model compounds (GC = glassy carbon, Cgr = graphite).

In 2014, Wu and Huang demonstrated electroreductive C−O bond cleavage in ethers in the presence of NaBH_4_ (Figure 6, top).^[65]^ A range of diaryl and alkyl aryl ethers, including lignin model compounds, formed phenolic products along with the corresponding deoxygenated aliphatic or aromatic compounds under galvanostatic conditions. Upon reduction of a fluorinated aromatic substrate, the desired reaction was accompanied by C−F bond cleavage. It was proposed that single‐electron reduction of the substrate leads to the corresponding anion‐radical that decomposes to a phenolate and a C‐radical, which reacts with a hydrogen donor to furnish the hydrocarbon product.[^18^, ^56^, ^57^, ^64^]

Electroreductive C−O bond cleavage in alkyl aryl ethers was recently reported by Stephenson and co‐workers (Figure 6, bottom).^[66]^ Here, electroreduction of oxidized β‐O‐4 lignin model compounds furnished phenols and ketones at an applied potential of −2.3 V vs SCE, while a lower applied potential (−2.7 V vs SCE) promoted formation of phenol and pinacol products. It was proposed that initial cathodic electron transfer to the substrate leads to a ketyl anion‐radical that decomposes to phenol and ketone upon a second electron transfer/protonation sequence. Under more reducing conditions, the ketone product is reduced to the corresponding ketyl radical, which dimerizes to form the pinacol product.

**Figure 7:**
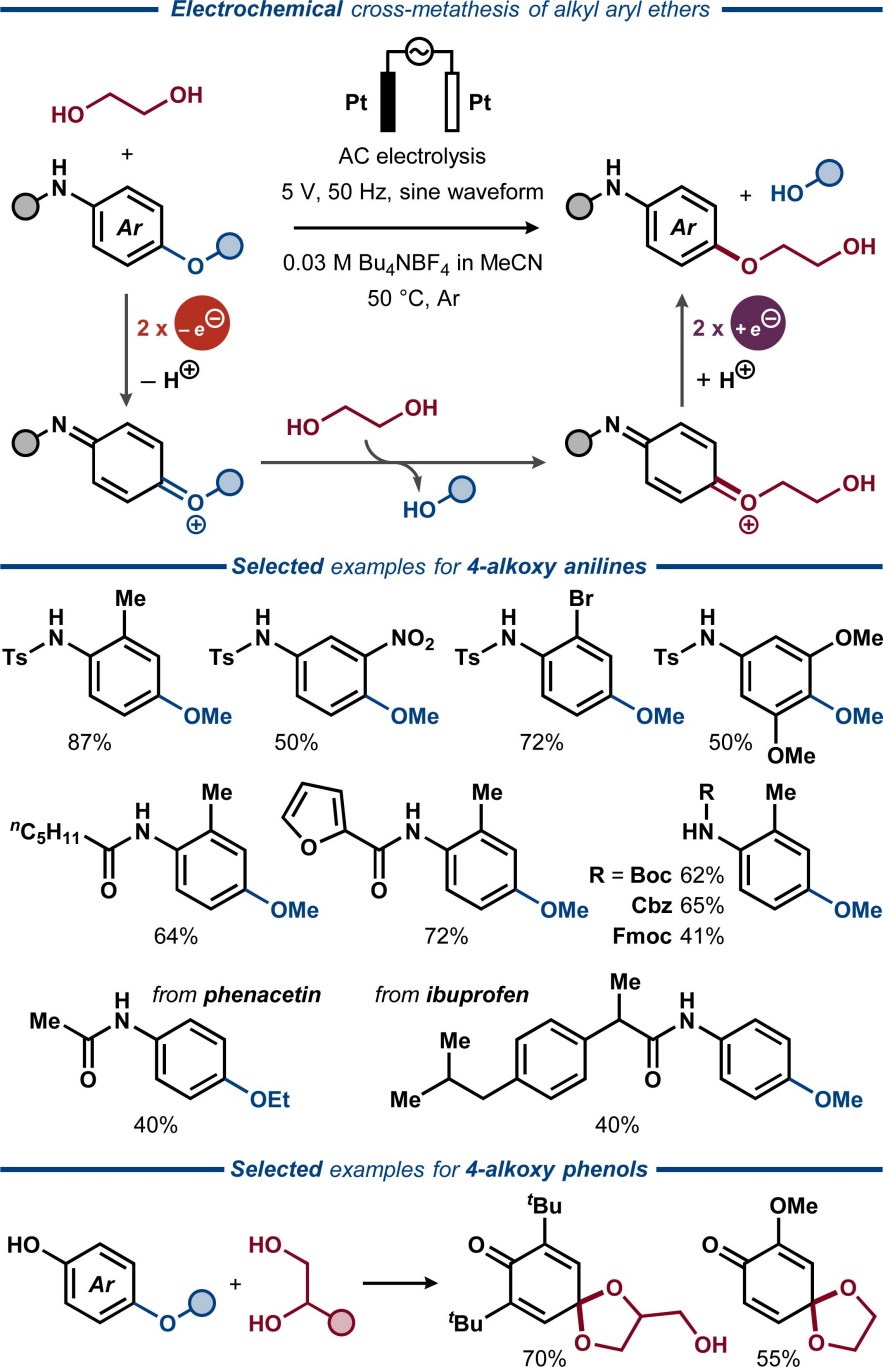
Electrochemical cross‐metathesis of aryl alkyl ethers and aliphatic diols, enabled by alternating current (AC) electrolysis.

Recently, Huang, Yuan, Lei, and co‐workers disclosed an electrochemical protocol employing alternating current (AC) electrolysis for cross‐metathesis between aryl alkyl ethers and aliphatic alcohols (Figure 7).^[67]^ The reaction was proposed to proceed through a stepwise two‐electron oxidation of the substrate to an imino quinone‐type intermediate that reacts with the alcohol nucleophile to form the corresponding ketal. The subsequent elimination of methanol furnishes a second imino quinone intermediate, which delivers the final product upon two consecutive reductive electron transfer events.

**Figure 8:**
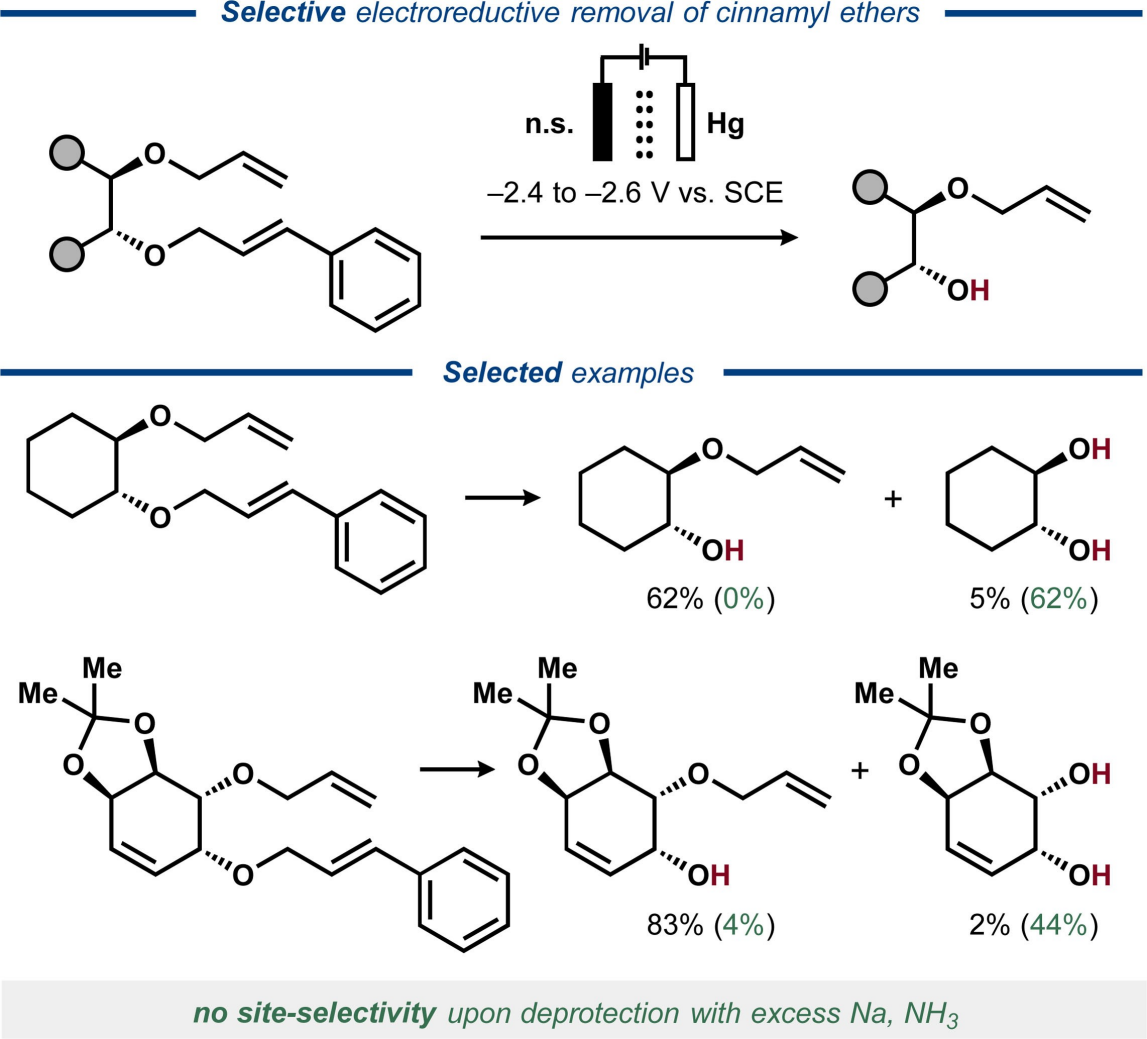
Selective electroreductive deprotection of alkyl cinnamyl ethers (n.s. = not specified).

In 1975, Santiago and Simonet reported that unsaturated conjugated ethers underwent either C−O bond cleavage or C=C bond reduction under electroreductive conditions.^[15]^ The selectivity of the reaction was found highly dependent on the nature of the substrate as well as on whether phenol was present as a proton source. In 2001, an electroreductive protocol was developed by Hudlicky and co‐workers for the cleavage of alkyl cinnamyl ethers in conduritol derivatives to afford the corresponding aliphatic alcohols. The authors observed higher selectivities with their electrochemical protocol compared to what they achieved under chemical Birch‐like conditions (Figure 8).^[68]^ Deprotection of cinnamyl ethers was also demonstrated under similar conditions in the presence of benzyl ethers and cinnamyl‐substituted amines, while bromides were reductively removed along with the cinnamyl group.^[69]^ Furthermore, cinnamyl esters, carbamates and carbonates were removed under the same conditions.

**Figure 9:**
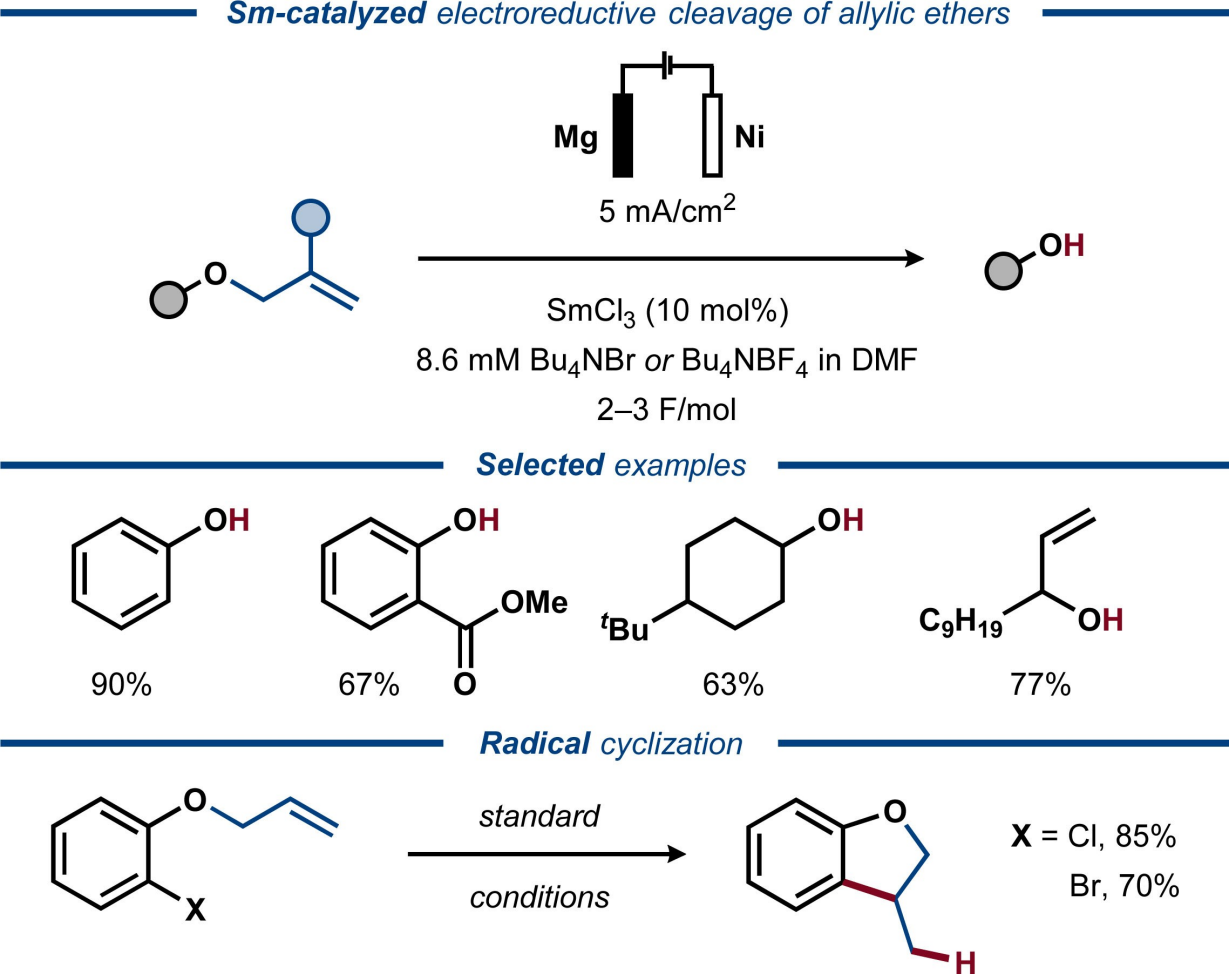
Electroreductive Sm‐assisted deallylation of allyl ethers.

In 1992, Duñach and co‐workers reported that the use of samarium complexes in substoichiometric amounts facilitates electroreductive deallylation of alkyl and aryl allyl ethers (Figure 9).^[70]^ The reactions were carried out under galvanostatic conditions with 10 mol % SmCl_3_ in an undivided cell, furnishing a variety of deallylated aromatic and aliphatic alcohols. In the absence of samarium, deallylation of certain substrates occurred at a higher overpotential, whereas aliphatic substrates failed to react. The authors speculated that divalent samarium species facilitate the electron transfers and/or assist the deallylation process by coordinating to the ether oxygen as a Lewis acid.^[71]^ In addition to deallylation of allyl ethers, removal of allyl esters was achieved under the electroreductive conditions, whereas methyl esters remained intact.

**Figure 10:**
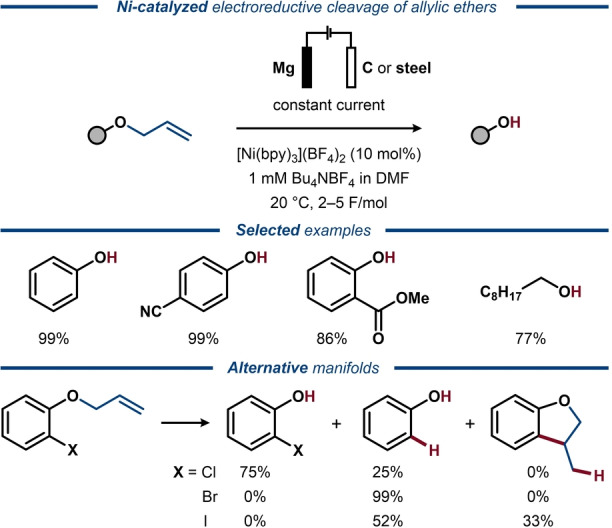
Electroreductive Ni‐catalyzed deprotection of allyl ethers.

It is well‐established that allyl ethers and alcohols can undergo C−O bond cleavage via oxidative addition to low‐valent transition metal catalysts to form η^3^‐allyl species,^[72]^ and electrochemically driven processes have been disclosed.[^73^, ^74^] In the 1990s, Yasuhara and Sakamoto reported a two‐step method for stoichiometric reductive deprotection of aryl allyl ethers using electrogenerated nickel species.^[75]^ A related catalytic protocol was developed by Duñach and co‐workers in 1995 for selective cleavage of allyl ethers derived from aromatic and aliphatic alcohols (Figure 10).^[76]^ Here, electrolysis in the absence of the catalyst resulted in mixtures of products, while esters, aromatic chlorides and nitriles remained intact under the catalyzed reaction conditions and iodide and bromide substituents were removed. Enol ethers were found unreactive, suggesting that the Ni‐catalyzed protocol proceeds via an η^3^‐allyl intermediate and not via double bond isomerization. Mechanistic studies supported this proposal and indicated that Mg^2+^ ions, formed upon oxidation of the sacrificial anode, facilitated the turnover of the Ni catalyst.^[77]^

**Figure 11:**
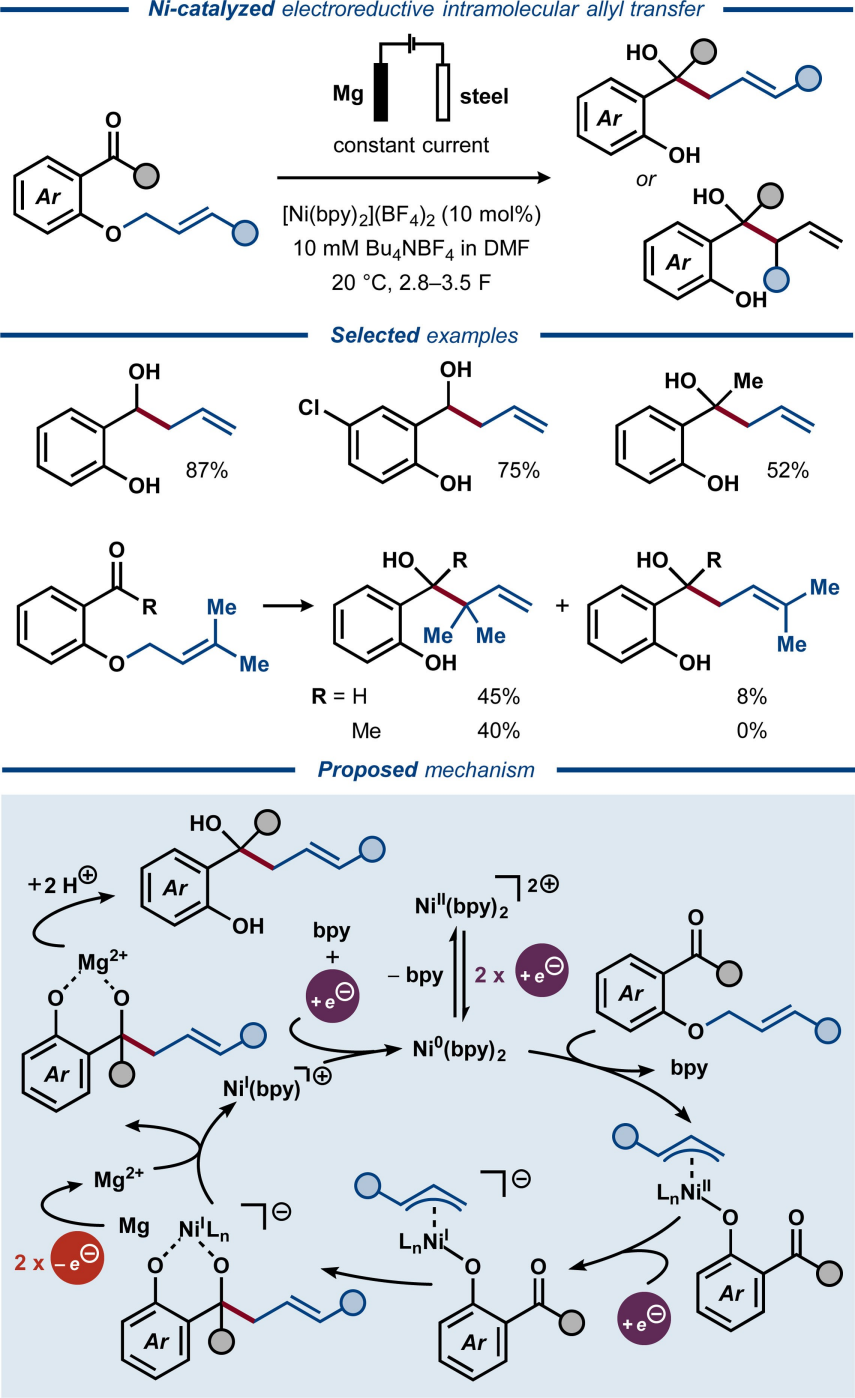
Electroreductive Ni‐catalyzed intramolecular allyl transfer.

In the late 1990s, the deallylation strategy was extended by Duñach to allyl transfer reactions using both Pd‐ and Ni‐catalysis.[^78^, ^79^] Initial metal‐catalyzed electrochemical cleavage of the allyl ether moiety in *o*‐(allyloxy)benzaldehydes and *o*‐(allyloxy)acetophenones was followed by intramolecular allyl transfer to the carbonyl to afford homoallylic alcohols. The ratio of branched to linear homoallylic alcohol products was found substrate‐dependent, with an overall preference for the linear products (Figure 11). A separate study correlated the regioselectivity with the nature of the formed ions at the sacrificial anode.^[80]^ Interestingly, while Mg^2+^ ions were found to facilitate catalyst regeneration, an improved regioselectivity was obtained in their absence. Mechanistic studies suggested that the reaction proceeds via Ni^0^ insertion into the C−O bond of the allyl ether to form an η^3^‐allylic‐Ni^II^ intermediate,^[81]^ which undergoes single‐electron reduction to Ni^I^, followed by rapid intramolecular allyl transfer to the carbonyl group. Notably, the cleavage of the allylic C−O bond with subsequent allyl transfer was observed at −1.75 V vs. SCE using the Ni catalyst, while direct electroreductive cleavage of allylic ethers requires considerably lower potentials (<2.4 V vs. SCE).^[68]^ This striking difference illustrates how catalysis can benefit electrosynthesis by enabling milder conditions for improved functional group tolerance. The Ni‐catalyzed protocol was later extended to propargylic substrates,[^82^, ^83^] as well as intramolecular allyl transfer to adjacent α‐halides.^[84]^ Similarly, intermolecular allyl transfer from allyl phenyl ether to ethyl 4‐bromobenzoate has been reported using an electrogenerated Co^I^ catalyst.^[85]^

**Figure 12:**
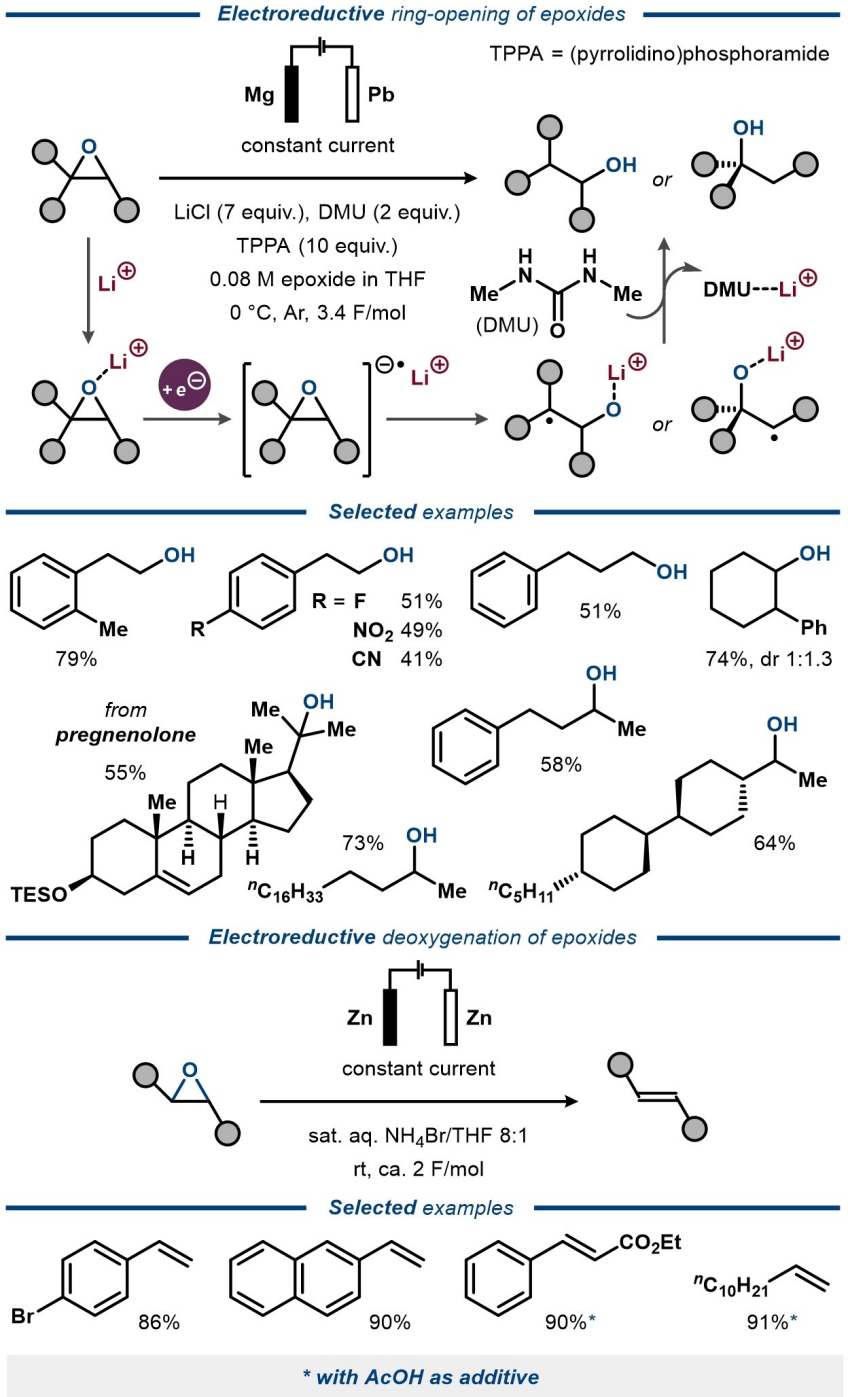
Electroreductive transformations of epoxides.

Electroreduction of epoxides to mixtures of alcohols, alkenes and alkanes has been known for decades.^[86]^ A selective direct electrolysis protocol for epoxide ring‐opening to primary, secondary, and tertiary alcohols was recently reported by Qi, Lu and co‐workers using ureas as hydrogen donors (Figure 12, top).^[87]^ Here, the regioselectivity of the reaction was controlled by the innate reactivity of the substrate, favoring anti‐Markovnikov products for aryl‐substituted epoxides and Markovnikov products for alkyl‐substituted epoxides. This reactivity difference was rationalized to stem from the thermodynamic stability of benzyl radical intermediates in the former substrate class and the kinetic preference for terminal hydrogenation in the latter. A complementary protocol for ring‐opening of aryl‐substituted epoxides to alkenes under aqueous conditions was reported by Huang and co‐workers (Figure 12, bottom).^[88]^

**Figure 13:**
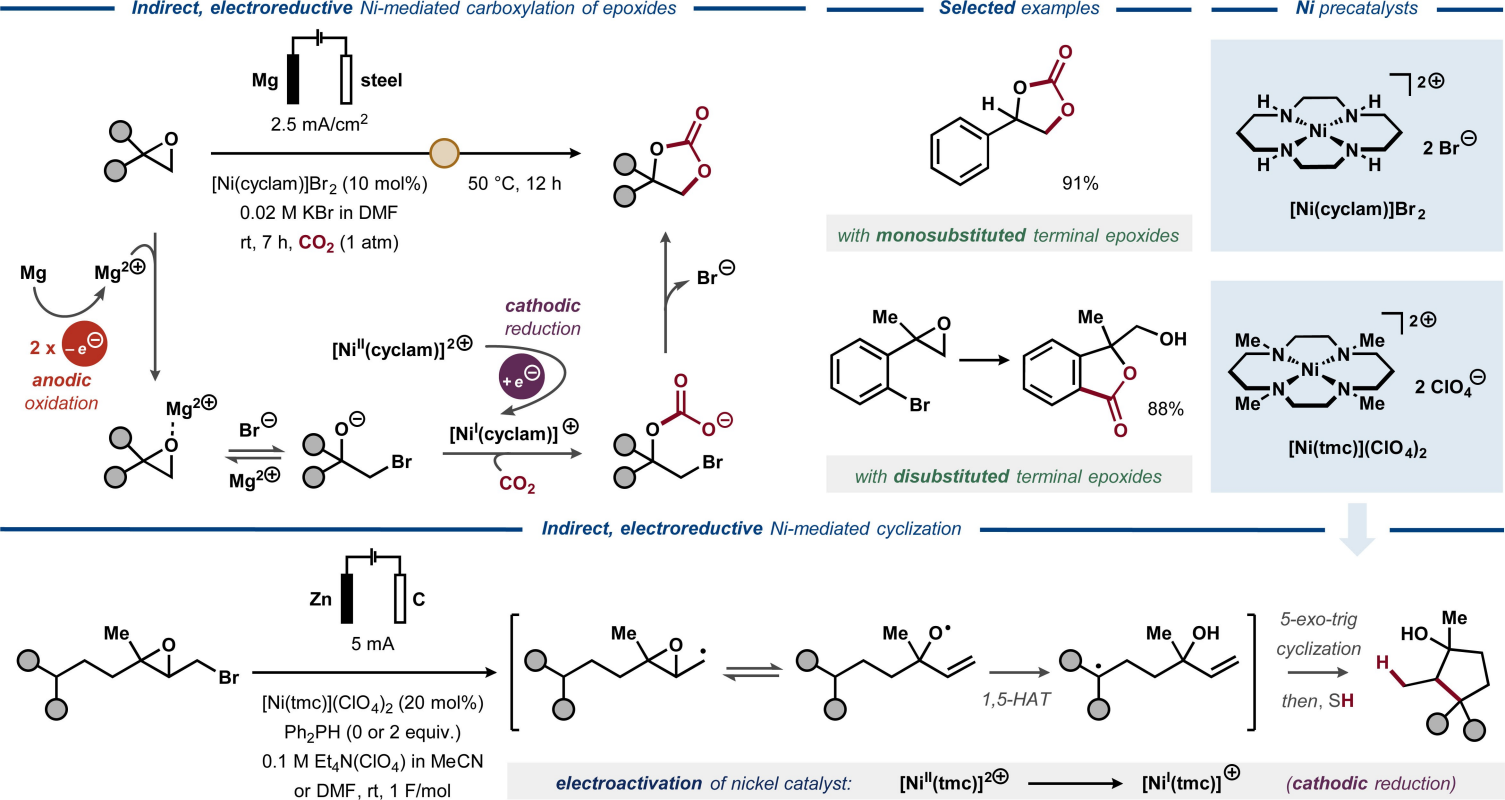
Electroreductive Ni‐catalyzed carboxylation (top) and cyclization (bottom) of epoxides.

Electrochemical fixation of CO_2_ is a known method for creating new C−C or C−heteroatom bonds and is predominantly used for accessing carboxylic acids in an *electrocarboxylation* process.^[89]^ In the late 1990s and early 2000s, electrocarboxylation of epoxides to form cyclic carbonates was reported by Duñach and co‐workers,^[90]^ utilizing electrogenerated Ni^I^‐complexes as catalysts (Figure 13, top).^[90a]^ The epoxide ring‐opening was proposed to proceed via nucleophilic attack of a bromide ion, facilitated by Lewis acidic Mg^2+^ ions that form in the reaction mixture upon dissolution of the sacrificial anode. In parallel, the electroreductively formed Ni^I^ species activates CO_2_ and mediates the subsequent carboxylation, resulting in ring closure upon bromide elimination.[^24^, ^90b^, ^90c^] The transformation proceeded with good yields and selectivities for aromatic, benzylic and aliphatic epoxides. The method was also applicable for terminal disubstituted epoxides, resulting in hydroxybenzolactones, although via a different reaction pathway.^[90b]^

Complementary to Duñach's strategy, Ozaki and co‐workers developed a Ni‐catalyzed protocol for reductive transformation of brominated epoxides in 1997 (Figure 13, bottom).^[91]^ Here, the electrogenerated Ni^I^ catalyst was used to induce C−Br bond cleavage to furnish a C‐radical that rearranges to the corresponding allyloxy radical. This O‐radical participates in intramolecular 1,5‐hydrogen atom transfer (1,5‐HAT) to furnish a second C‐radical, which furnishes an open‐chain allylic alcohol product upon H‐atom abstraction from the hydrogen donor diphenyl phosphine (Ph_2_PH). Alternatively, intramolecular addition of C‐radical to the terminal double bond of the substrate furnishes cyclopentanol products. In the absence of the hydrogen donor, dimerization of the cyclopentanols was observed.

**Figure 14:**
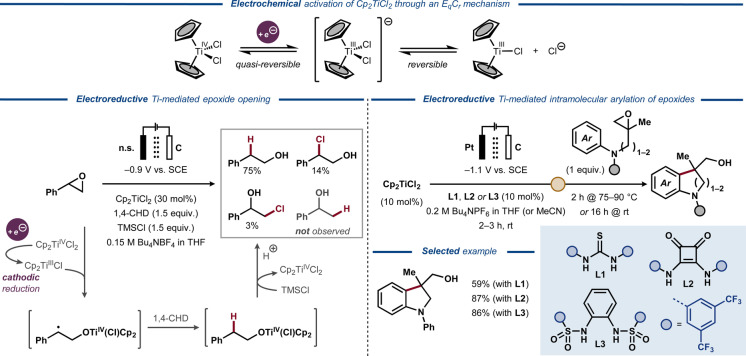
Electroreductive Ti‐catalyzed ring‐opening of epoxides. Top: Electroreductive activation of titanocene dichloride. Bottom left: Electroreductive, titanocene‐mediated epoxide opening. Bottom right: Electroreductive titanocene‐mediated radical arylation of epoxides.

The Nugent–RajanBabu reagent Cp_2_Ti^III^Cl can be generated from Cp_2_Ti^IV^Cl_2_ with chemical reductants, such as organometallic complexes, or by electrochemical electron transfer through an E_q_C_r_ mechanism (Figure 14, top).^[92]^ The equilibrium between the resting state of the catalyst ([Cp_2_Ti^IV^Cl_2_]^−^) and the active species (Cp_2_Ti^III^Cl) can be altered in favor of the active catalyst by abstraction of the chloride anion.^[93]^ In 2011, electrochemically induced epoxide ring opening to form various alcohols was described by Magdesieva and Nikitin, using Cp_2_Ti^III^Cl as catalyst (Figure 14, bottom left).^[94]^ In a divided cell under potentiostatic conditions, the tetravalent titanocene precatalyst was reduced to the active trivalent Ti complex. The coordination of Cp_2_Ti^III^Cl to the epoxide substrate was proposed to induce the ring opening and the formation of a C‐radical that reacts with the H‐donor 1,4‐cyclohexadiene (1,4‐CHD). Finally, Ti−O bond cleavage was induced by trimethylsilyl chloride, with the resulting silylated alcohol being hydrolyzed during acidic workup to furnish the primary alcohol product. In the same vein, Gansäuer and co‐workers studied the electroactivation of titanocene dichloride and dibromide for intramolecular radical arylation of epoxides (Figure 14, bottom right).^[93]^ Addition of thiourea (**L1**), squaramide (**L2**) or bissulfonamide (**L3**) was found to promote the reversible dissociation of a halide ligand from the initially formed anionic Ti^III^ complex, facilitating the formation of the catalytically active Cp_2_TiCl or Cp_2_TiBr and affording an anodic shift of the reduction peak potential of the catalyst. In the presence of the more accessible catalyst Cp_2_TiCl_2_, additives **L2** and **L3** delivered the expected product in higher yields compared to **L1**.

**Figure 15:**
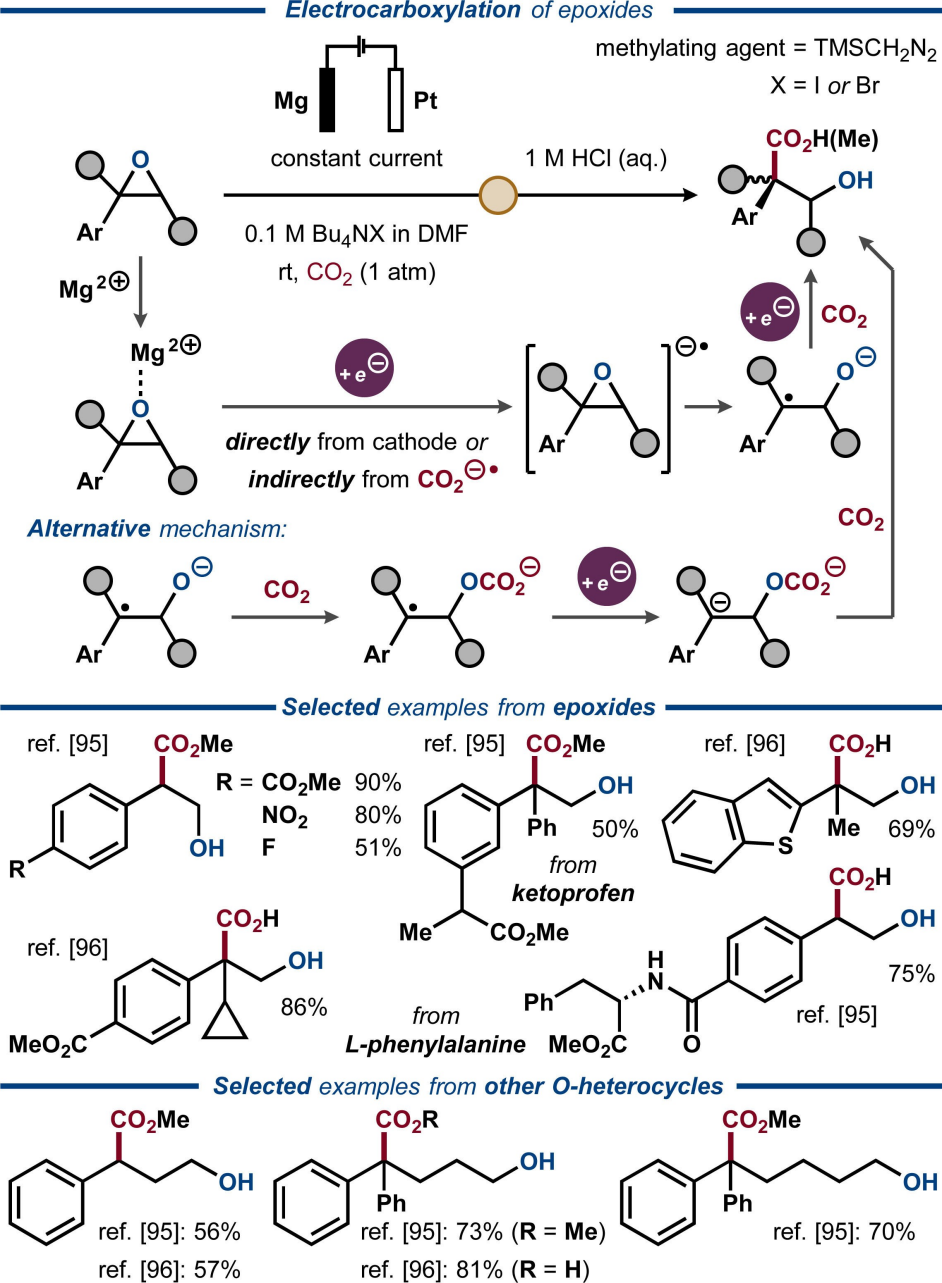
Direct electrocarboxylation of epoxides.

Direct electrocarboxylation of epoxides and other cyclic ethers (2–5 carbons) to hydroxy acids was recently reported by the groups of Qiu^[95]^ and Zhang^[96]^ (Figure 15). Both groups proposed that single‐electron transfer from the cathode to the cyclic ether results in formation of an anion‐radical intermediate, followed by ring‐opening via C−O bond cleavage and formation of a benzylic C‐radical. A second electron transfer furnishes the corresponding benzylic carbanion that reacts with CO_2_ and furnishes the hydroxy acid upon acidic workup. In both cases, enantiomerically pure epoxides were fully racemized under the reaction conditions. In contrast, a protocol for direct electrocarboxylation of enantiopure epoxides to chiral carbonates was recently reported by Wang, Lu and co‐workers.^[97]^

## 4 Electrochemical C−O Bond Activation in Carboxylic Esters

Carboxylic acids and their derivatives are highly abundant among natural and synthetic organic compounds. Oxidative activation of carboxylic acids through Kolbe electrolysis in the 19^th^ century^[98]^ marked the dawn of preparative electrosynthesis and spurred a plethora of decarboxylative methods proceeding through homolytic C−C bond cleavage.^[99]^ While deoxygenative electrochemical C−O bond activation of alcohols is limited to activated substrates, analogous activation of acylated alcohol derivatives proceeds more readily. For example, chemical radical‐mediated reduction of esters via two consecutive one‐electron reductions was realized already in the beginning of the 20^th^ century in the Bouveault–Blanc reaction, using sodium metal as reductant.^[100]^ Electrochemical reductive C−O bond activation has been described in a number of electroanalytical studies starting from the middle of the 20^th^ century. Here, one‐electron reduction of aromatic esters in aprotic media was shown to furnish relatively long‐lived anion‐radicals, which were proposed to undergo either C(O)−O bond cleavage to an acyl radical and alkoxide, or C(O)O−C bond cleavage to a corresponding carboxylate and a C‐radical. As detailed below, only the latter reactivity mode was successfully applied in a preparative setting.

**Figure 16:**
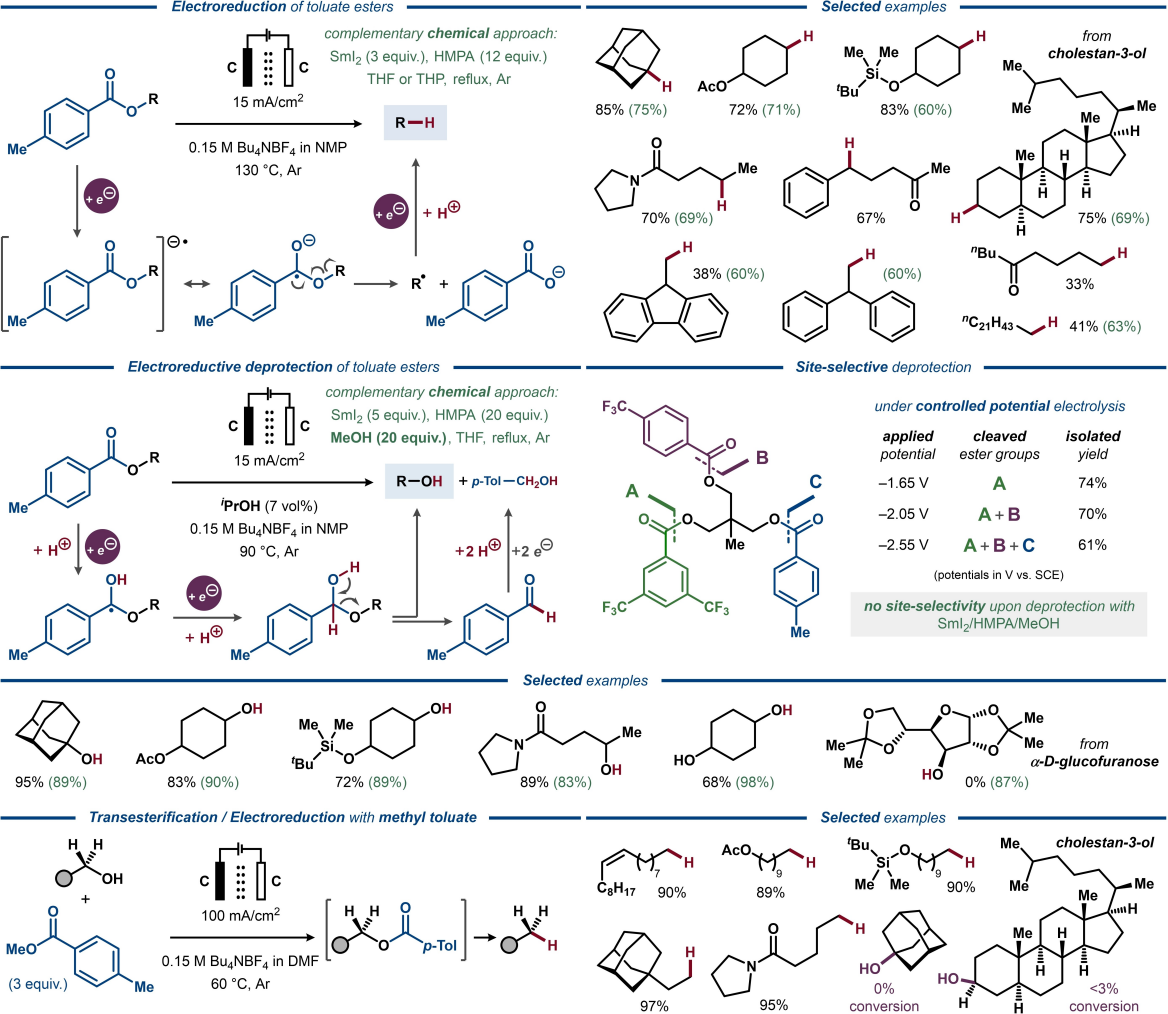
Electrochemical C−O bond activation in toluate esters. Top: Electroreductive deoxygenation of toluate esters in aprotic media. Middle: Electroreductive deprotection of toluate esters in protic media. Bottom: Electrochemically induced transesterification–deoxygenation of toluate esters.

A landmark series of studies detailing homolytic C(O)O−C bond cleavage in aromatic esters was conducted by Lam and Markó,^[101]^ complemented by electroanalytical studies by others.[^102^, ^103^] While examining a more practical alternative to the Barton–McCombie reaction,[^10^, ^104^] Lam and Markó studied the electrochemical behavior of aliphatic benzoates at low potentials in aprotic media,^[105]^ demonstrating an E_r_C_i_ mechanism for their one‐electron reduction. While only a small difference in the reduction potentials was found for a series of aliphatic benzoates (*E*_1/2_≈−2.3 V vs. SCE),^[24]^ the rate of decomposition of the anion‐radical (C_i_ step) strongly depended on the stability of C‐radical formed upon C(O)O−C bond cleavage. Accordingly, the rate of decomposition increased by one order of magnitude when going from primary to secondary and tertiary radicals, while an even more pronounced rate enhancement was observed for allyl benzoate. When employing *p*‐toluate ester as the activating group, the authors achieved efficient reductive deoxygenation of a number of aliphatic alcohols under chemical conditions (Figure 16, top).^[105]^

In a subsequent study, the reductive deoxygenation was further demonstrated by preparative galvanostatic electrolysis in a divided cell at 130 °C (Figure 16, top).^[106]^ The aliphatic products derived from tertiary and secondary alcohols were isolated in good to high yields, while esters of primary alcohols were less efficient. Unprotected and silylated alcohol, aliphatic ester and amide functionalities were well‐tolerated under the developed conditions. Notably, most substrates containing a potentially reducible ketone functionality provided the deoxygenated products with the ketone intact.

Electroanalytical studies of ethyl *p*‐toluate in the presence of methanol demonstrated a profound influence of the availability of protons on the underlying electrochemical processes.[^107^, ^108^] Using cyclic voltammetry (CV), the originally observed quasi‐reversible wave of *p*‐toluate ester in aprotic solvents was rendered fully irreversible upon addition of methanol, which was explained by fast and irreversible protonation of the initially formed anion‐radical. Akin to the Bouveault–Blanc reaction, the second one‐electron/one‐proton reduction step could then take place under preparative conditions, furnishing an unstable hemiketal intermediate, which spontaneously decomposes to the corresponding alcohol and aldehyde constituents. Subsequently, the aldehyde is engaged in another series of one‐electron reduction–protonation steps, producing the corresponding alcohol. This sequence was successfully realized for both SmI_2_‐ and electrochemically promoted reduction of a range of *p*‐toluate esters with methanol and 2‐propanol as the respective protic additives (Figure 16, middle). The electrochemically promoted deprotection of *p*‐toluate esters proved highly efficient for the functionalized substrates employed in the previously disclosed electrochemical C−O bond cleavage reaction.^[106]^ Notably, substrates featuring multiple substituted benzoate esters with varying reduction potentials could be selectively deprotected under constant potential electrolysis conditions, while no such selectivity was observed for the complementary SmI_2_‐promoted deprotection reaction.

The electroreductive deoxygenation of *p*‐toluate esters was expanded by Lam and Markó to encompass a transesterification–deoxygenation approach, allowing deoxygenation of primary aliphatic alcohols in the presence of an excess of methyl *p*‐toluate (Figure 16, bottom).^[109]^ Under conditions similar to the direct electroreductive deoxygenation of *p*‐toluate esters,[^106^, ^107^, ^108^] a variety of primary alcohols equipped with a range of functional groups furnished the corresponding deoxygenated products in excellent yields. Secondary and tertiary alcohols failed to deliver the deoxygenated products, which was rationalized as the result of increased steric hindrance that impedes the transesterification step.

The readily prepared carboxylic esters of oxalic acid have for long been known to be susceptible to single‐electron transfers and formation of C‐radicals upon release of CO_2_,[^110^, ^111^] and gave rise to numerous photoredox‐catalyzed deoxygenation reactions.^[112]^ The more electron‐deficient character of the oxalate esters manifests in significantly more anodic reduction potentials (*E*>−1.7 V vs. SCE)[^16^, ^24^] compared to benzoate esters (*E*_1/2_≈−2.3 V vs. SCE),^[105]^ which is attractive from a synthetic perspective. However, the strongly electrophilic nature of oxalate esters makes them more amenable to hydrolysis by adventitious or electrogenerated bases and to transesterification reactions, posing a significant challenge during electroreduction of oxalate esters on preparative scale.

**Figure 17:**
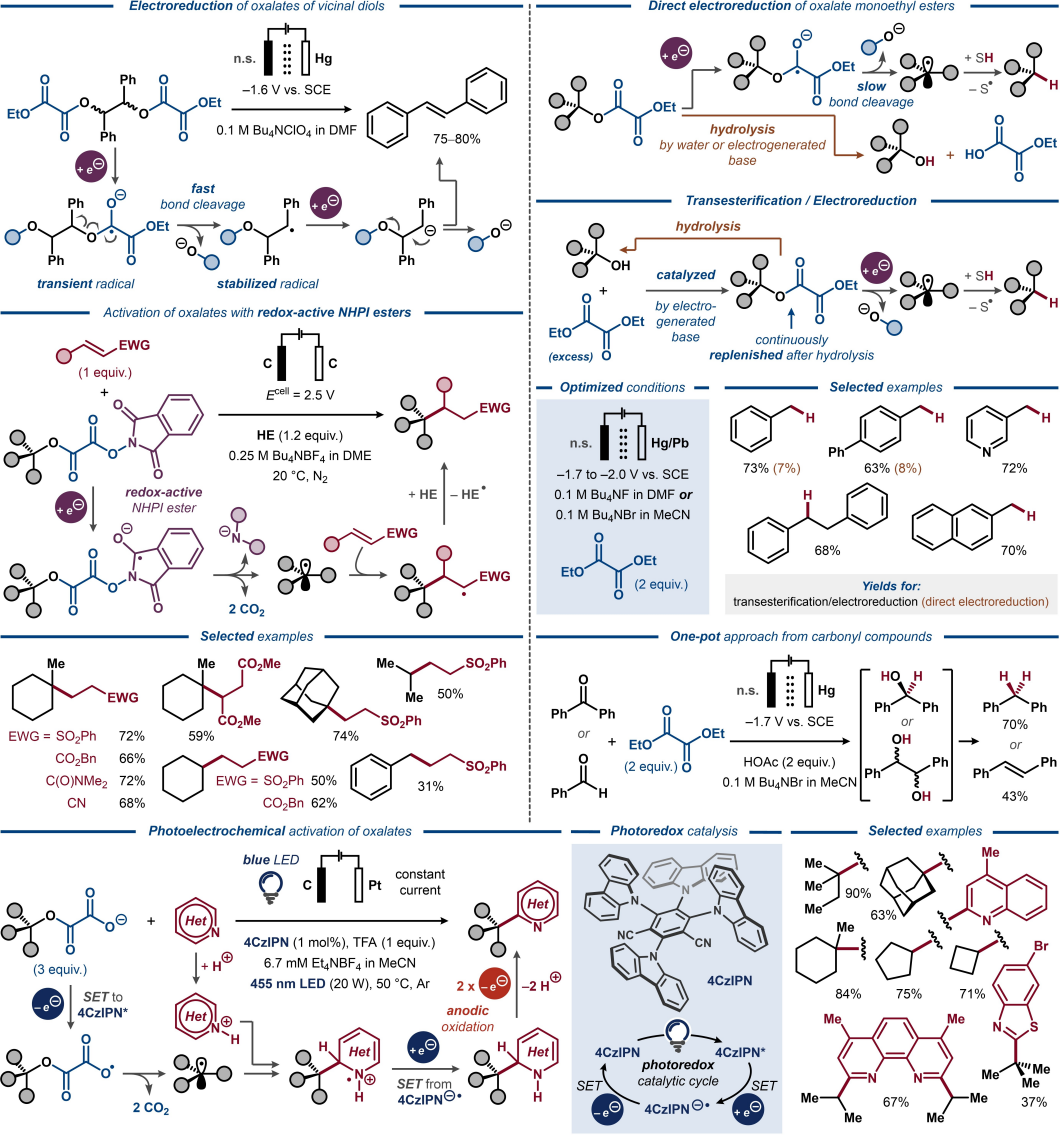
Electrochemical C−O bond activation in oxalate esters. Top left: Electroreductive deoxygenative desaturation of oxalate diester, n.s.=not specified. Top right: Electrochemically induced transesterification–deoxygenation of ethyl oxalate diesters. Middle left: Electroreductive functionalization of NHPI–oxalate esters. Bottom: Photoelectrochemical heteroarylation of oxalate monoesters.

The moderately negative reduction potential of oxalate esters was exploited by Utley and co‐workers for selective reduction of monoethyl oxalate diesters of vicinal diols to alkenes under electrochemical conditions (Figure 17, top left).^[16]^ Conducting preparative potentiostatic electrolysis at mildly negative potentials effectively suppressed overreduction of the alkene product, allowing effective conversion of hydrobenzoin monoethyl oxalate diesters to *trans*‐stilbene. The reaction was proposed to proceed through one‐electron reduction of the oxalate ester to furnish a C‐radical that undergoes another one‐electron reduction to a carbanion, followed by elimination of monoethyl oxalate anion concomitant with C=C bond formation. The same mechanism was proposed for the related electroreduction of α,α′‐diacetoxy stilbenes to diphenylacetylene as the sole product.[^113^, ^114^]

Similar to the reactivity observed for *p*‐toluate esters, the efficiency of the C(O)O−C bond cleavage in oxalate anion‐radical intermediates was found highly dependent on the stability of the resulting C‐radical.^[115]^ Unlike the hydrobenzoin‐derived monoethyl oxalate diesters (Figure 17, top left), electroreduction of monoethyl oxalate diesters of monoalcohols gave rise to less stabilized C‐radicals, precluding fast homolytic C−O bond cleavage of the initially formed oxalate anion‐radical.[^116^, ^117^] As a result, the latter becomes amenable to hydrolysis by adventitious water or electrogenerated bases, leading to formation of an alcohol as side‐product. Circumvention of this side‐reaction was achieved by addition of an excess of diethyl oxalate (Figure 17, top right), analogous to the abovementioned transesterification protocol for deoxygenation of primary alcohols.^[109]^ Under such conditions, a range of benzylic alcohols could successfully engage in the reductive transesterification–deoxygenation sequence.[^116^, ^117^] Furthermore, the benzylic alcohol substrates could be prepared by electroreduction of related carbonyl compounds in a one‐pot fashion.^[117]^ A related one‐pot procedure was found applicable for reductive deoxygenation and dimerization of biomass‐based furfural derivatives.^[118]^

Recently, electrochemical generation of C‐radicals from mixed *N*‐hydroxyphthalimide (NHPI) oxalate esters followed by a Giese‐type radical addition to alkenes was demonstrated by Wang and co‐workers under potentiostatic conditions (Figure 17, middle left).^[119]^ The redox‐active NHPI ester is reduced at more anodic potentials (*E*_1/2_>−1.2 V vs. SCE) compared to the oxalate ester, producing a phthalimide‐localized anion‐radical, which readily eliminates a phthalimide anion and two CO_2_ molecules to afford the key C‐radical intermediate. Subsequently, this radical adds to a Michael acceptor and furnishes the Giese adduct upon abstraction of a H‐atom from a Hantzsch ester (HE). The sequence was successfully realized for a range of NHPI–oxalate esters formed from tertiary alcohols and a range of Michael acceptors, whereas substrates based on secondary and primary benzylic alcohols required elevated temperatures and increased cell potentials to furnish the desired Giese products in moderate yields. Despite the moderate performance and scope, this electroreductive system showcased a viable, metal‐free alternative to related transition metal‐catalyzed manifolds, where activation of oxalate diesters requires stoichiometric amounts of metal reductants.[^120^, ^121^, ^122^]

An unusual example of oxidative activation of oxalate monoesters was recently demonstrated by Xu under photoelectrochemical conditions (Figure 17, bottom).^[123]^ In this approach, the negatively charged oxalate functionality undergoes one‐electron oxidation by an excited‐state photoredox catalyst (4CzIPN*) to produce a carboxylate radical and the reduced photocatalyst (4CzIPN⋅^−^). The formed carboxylate radical undergoes elimination of two CO_2_ molecules, furnishing the key C‐radical intermediate, which is trapped by a protonated heteroarene. Subsequently, the resulting cation‐radical is reduced by 4CzIPN⋅^−^ to a 1,2‐dihydroheteroarene intermediate, which is rearomatized upon anodic oxidation to form the desired 2‐alkylsubstituted heteroaromatic Minisci‐type adduct. This photoelectrochemical route was applied for alkylation of a range of substituted heteroaromatic compounds, producing the desired products in moderate to excellent yields. Notably, oxalate monoesters of tertiary, secondary and even primary alcohols served as efficient radical precursors, in contrast to the abovementioned protocol utilizing the mixed NHPI–oxalate esters as starting material.^[119]^

Similar to the observations made for mediated electrolysis of ethers,[^75^, ^76^, ^77^, ^78^, ^79^, ^80^, ^81^, ^82^, ^83^, ^84^] Pd and Ni catalysis can facilitate the net reductive C−O bond cleavage in π‐activated carboxylic esters.^[73]^ While direct electrochemical activation of allylic carboxylate functionalities, such as carbonate,^[143]^ carbamate[^69^, ^143^, ^144^] and ester,^[69]^ requires low potentials (*E*<−2 V vs. SCE),^[124]^ reduction of allylic acetates has been found to be highly efficient at considerably more anodic potentials (*E*≈−1.3 V vs. SCE) in the presence of a Pd catalyst^[125]^ and the use of late transition metal catalysts has facilitated the electrochemical deprotection of allyl carbonates and carbamates.[^126^, ^127^]

**Figure 18:**
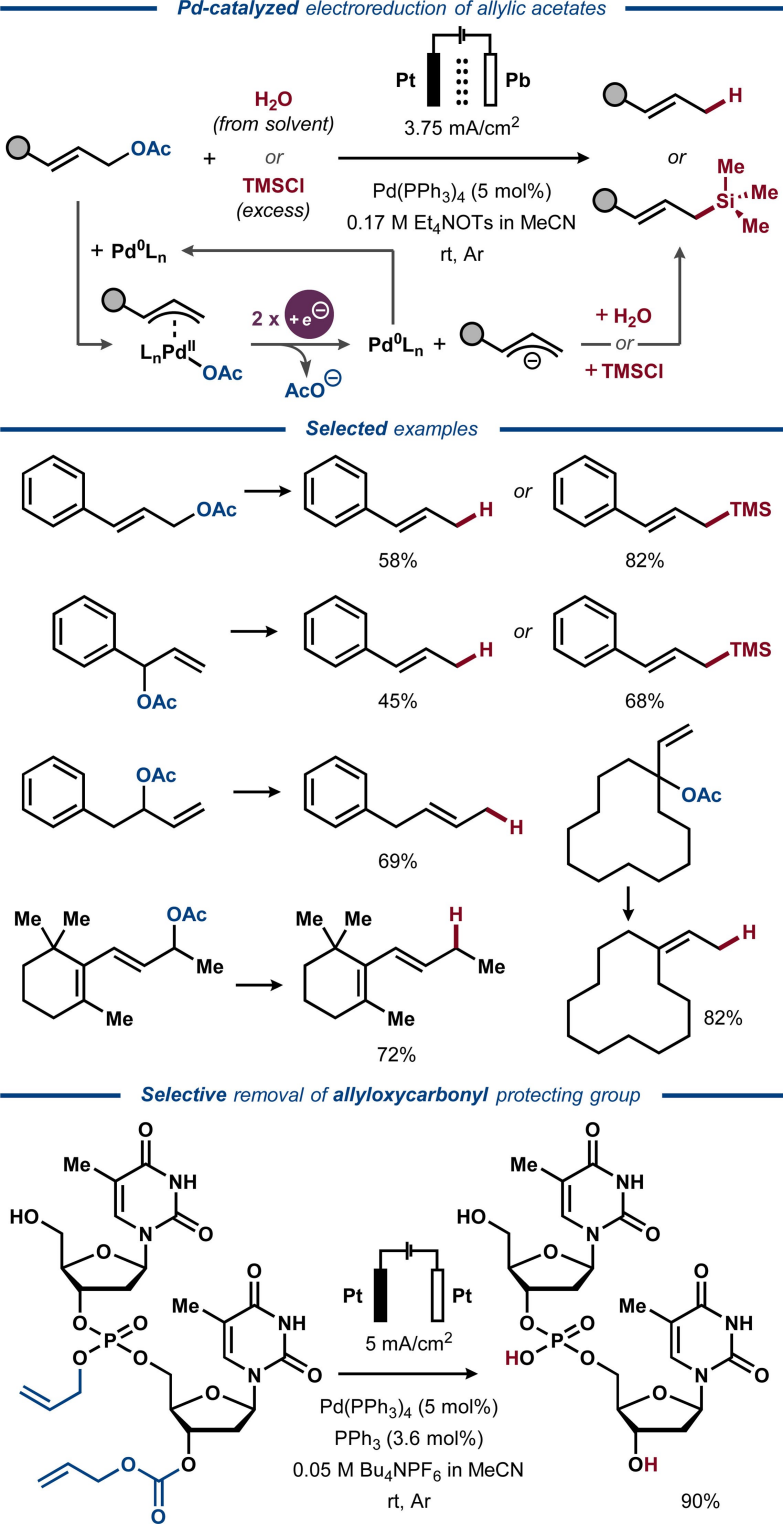
Electroreductive Pd‐catalyzed deoxygenation and silylation of allylic acetates.

Seminal work on Pd‐mediated electroreductive C−O activation of allylic acetates was disclosed by Torii and co‐workers in 1984 to afford deoxygenated products (Figure 18).^[128]^ Initially, a Pd^0^ catalyst undergoes oxidative addition to the allylic acetate, analogous to the Tsuji–Trost reaction,^[129]^ producing an η^3^‐allyl Pd^II^ complex. However, whereas the Tsuji–Trost reaction involves addition of a carbon nucleophile to the electrophilic η^3^‐allyl ligand, the η^3^‐allyl Pd^II^ complex in the Torii protocol is reduced at the cathode to liberate an allylic anion as the key reaction intermediate. Capture of this intermediate by an electrophile (H^+^ or TMSCl) affords the deoxygenated olefin product in good to excellent yields.

In contrast to the work of Torii, the classical electrophilic reactivity of η^3^‐allyl Pd^II^ species was demonstrated in an electrochemical protocol by Nonaka and Fuchigami and co‐workers in 1986.^[130]^ Here, the authors adopted a step‐wise procedure, where a carbon nucleophile with acidic protons (dimethyl malonate, fluorene or nitromethane) and a Pd^II^ precursor together with a phosphine ligand were first subjected to electroreductive conditions in the cathode compartment of a divided cell to form nucleophilic electrogenerated bases along with the Pd^0^ catalyst. In the second step, an allylic acetate was added to the reaction mixture and allowed to stir in the absence of electrical bias to furnish the deoxygenated C−C coupling product. Curiously, not only efficiency of the reaction, but also regioselectivity of the nucleophile addition to 3,3‐disubstituted allyl acetates were found to be highly dependent on the nature of the electrolyte cation.

**Figure 19:**
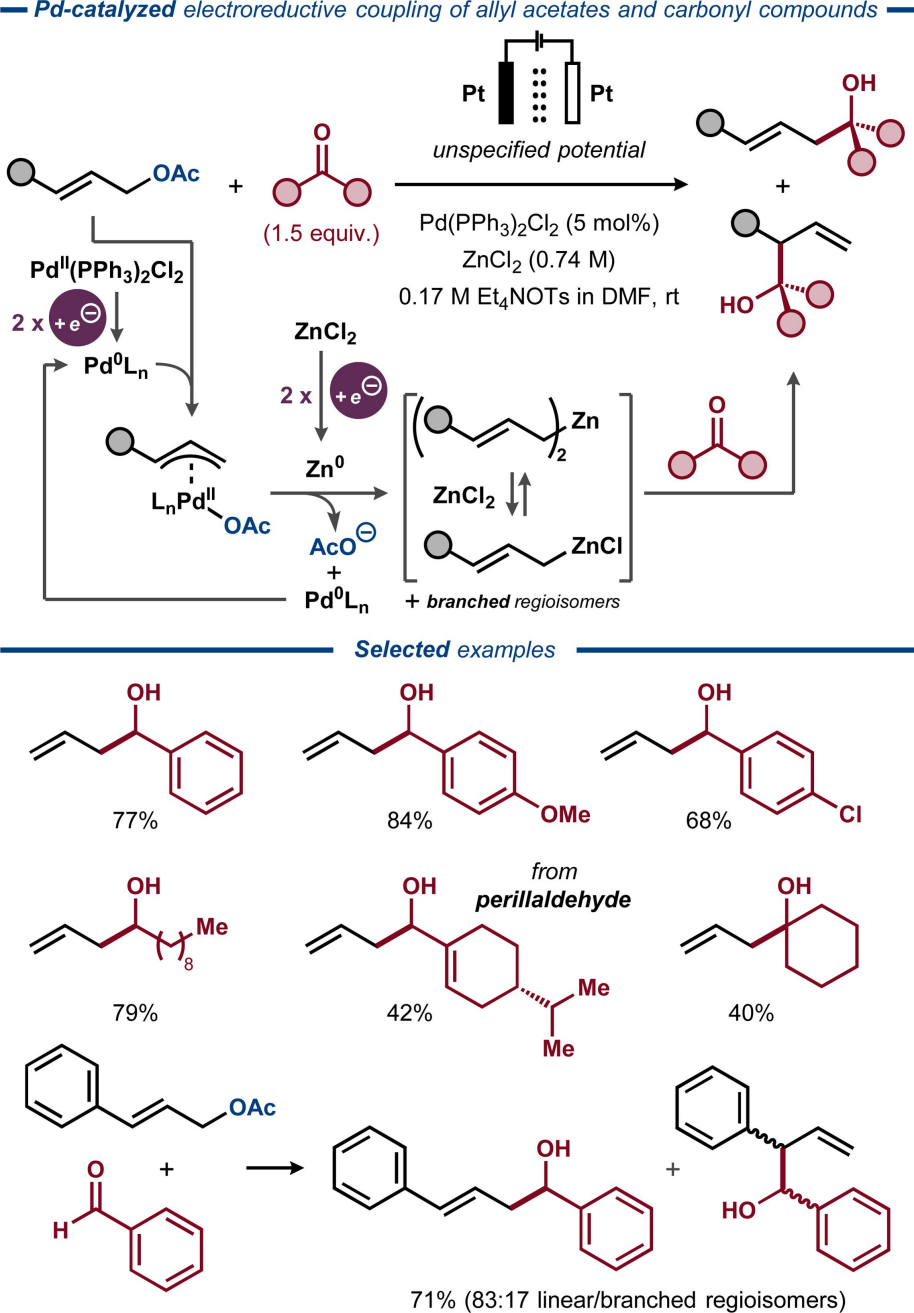
Electroreductive Pd/Zn‐catalyzed activation of allylic acetates for intermolecular coupling with carbonyl compounds.

In 1989, Qiu and Wang extended the scope of electrophiles compatible with Pd‐catalyzed electroreductive activation of allylic acetates.^[131]^ The electrocatalytic system utilized similar electroreductive conditions to those in the seminal report by Torii,^[128]^ but was made compatible with carbonyl electrophiles using ZnCl_2_ as a co‐catalyst. Based on earlier mechanistic studies,^[125]^ the η^3^‐allyl Pd^II^ species was proposed to react with the nascent Zn^0^ metal, generated on the cathode surface by electroreduction of ZnCl_2_, furnishing diallyl‐ or allylzinc species as key intermediates that react with the carbonyl electrophiles to form the homoallylic alcohol products (Figure 19). Coupling of allyl alcohol to a range of aromatic aldehydes proceeded with high efficiency, while aliphatic perillaldehyde and cyclohexanone provided the desired homoallylic alcohols in lower yields. Substituted allylic alcohols provided the desired alcohol products as a mixture of regioisomers.

**Figure 20:**
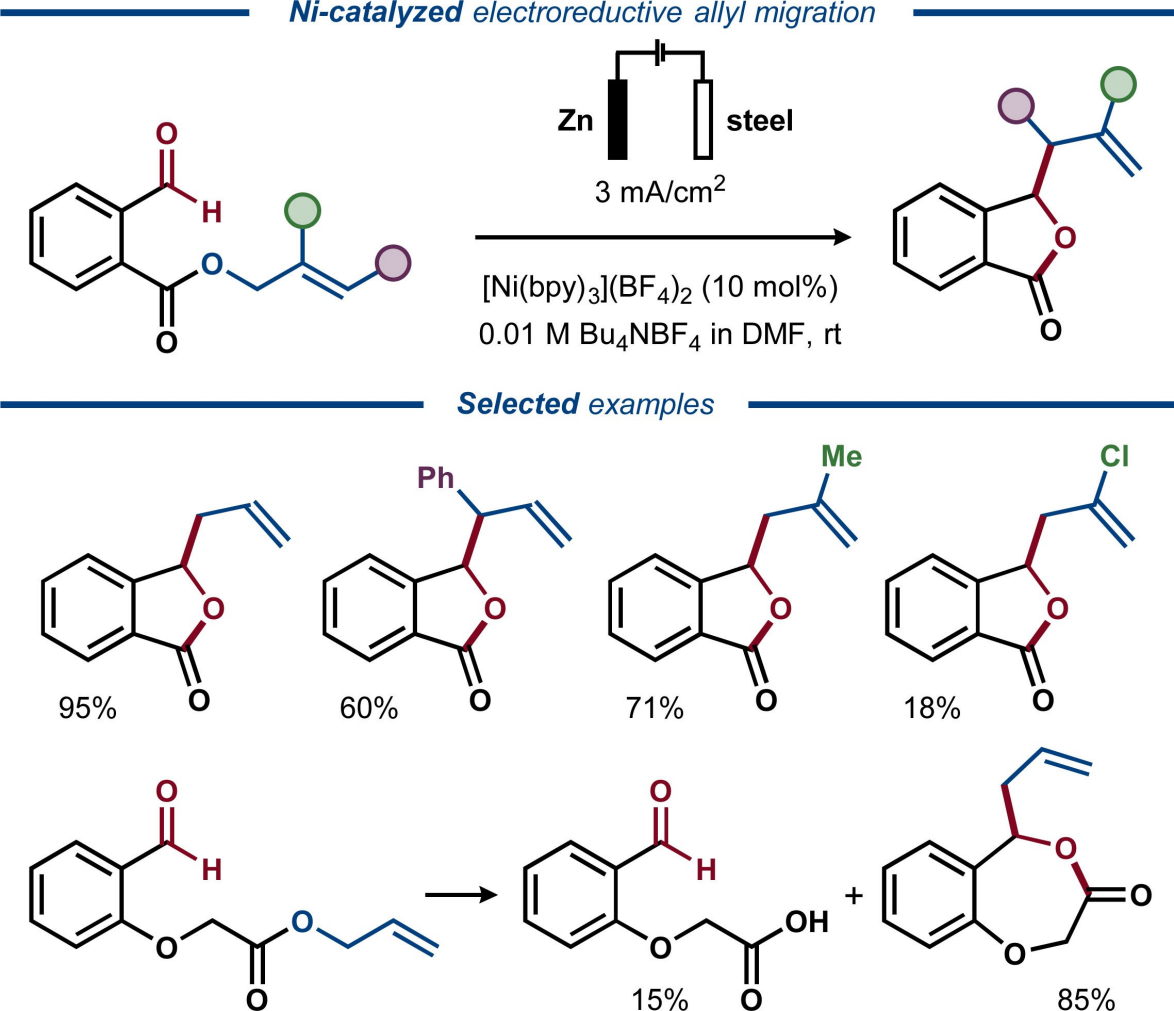
Electroreductive Ni/Zn‐catalyzed activation of allylic esters for intramolecular coupling with aldehydes.

In the early 2000s, a related intramolecular transformation utilizing Ni instead of Pd catalysis was demonstrated by Duñach for the conversion of allylic esters of *ortho*‐formyl benzoic acid to the corresponding allyl‐substituted benzolactones in good yields, including a seven‐membered lactone extended by a glycolic acid residue (Figure 20).^[132]^ Similarly, a protocol for intramolecular allyl transfer in allylic β‐keto esters was developed.^[133]^

**Figure 21:**
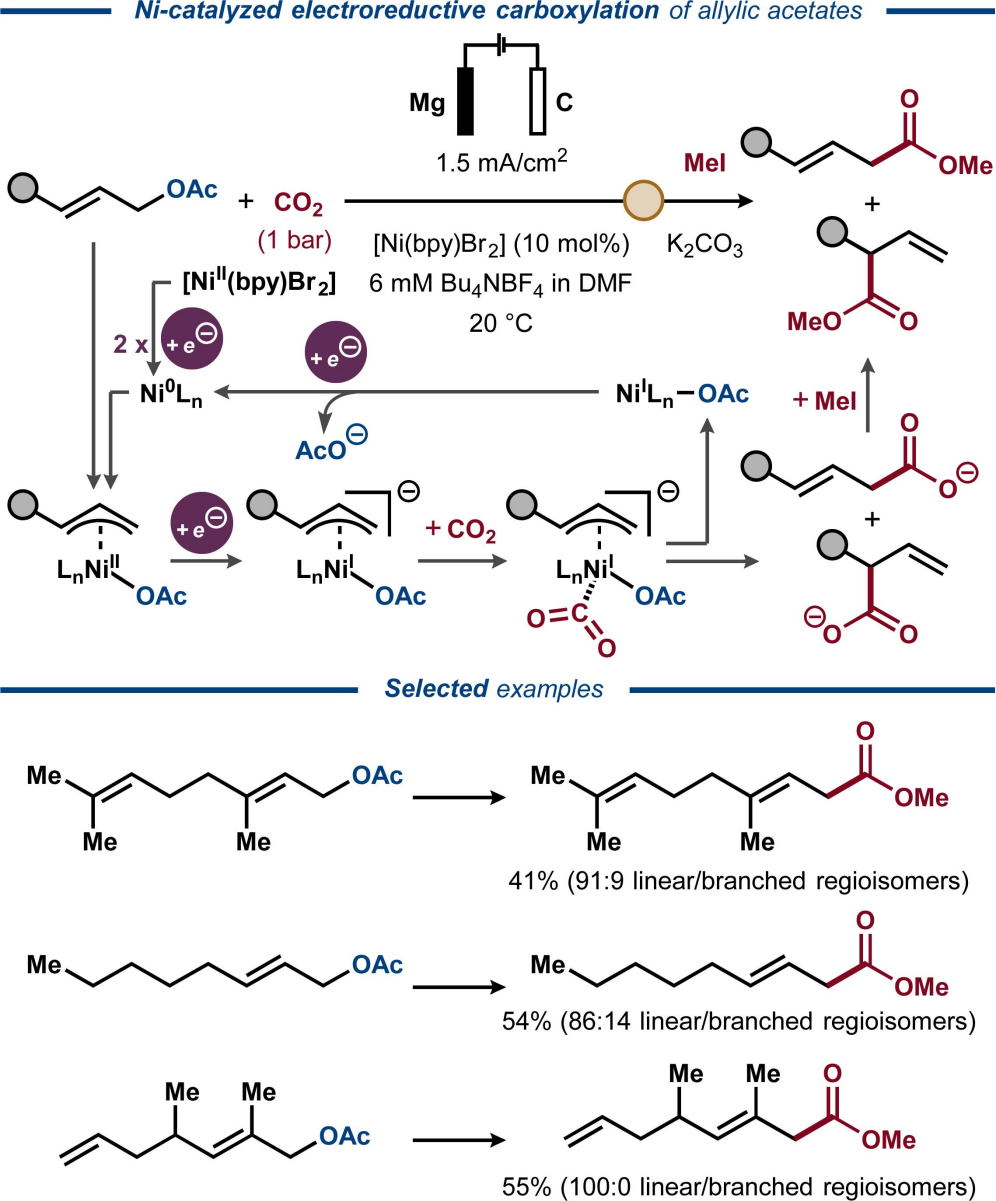
Electroreductive Ni‐catalyzed carboxylation of allylic acetates.

In 2011, deoxygenative electrocarboxylation of aliphatic allylic acetates with CO_2_ was demonstrated under Ni‐catalyzed reductive conditions by Duñach and co‐workers (Figure 21).^[134]^ Here, the model geranyl acetate substrate was converted to a carboxylic acid in 41% yield as a mixture of two regioisomers (91 : 9 linear/branched), while also producing an alkene by‐product in 59% yield. The other aliphatic allylic acetates provided the carboxylated products in similar yield and regioselectivity, with the exception of a more sterically hindered 2,4‐dimethylsubstituted allylic acetate, which provided the linear product as a single regioisomer. The method was extended to aliphatic allylic carbonates, delivering the carboxylated products with similar yields and regioselectivities. Mechanistic studies suggested that C−C bond formation between CO_2_ and the Ni‐coordinated allylic species is onset by cathodic one‐electron reduction of the η^3^‐allyl Ni^II^ complex to the Ni^I^ state.

**Figure 22:**
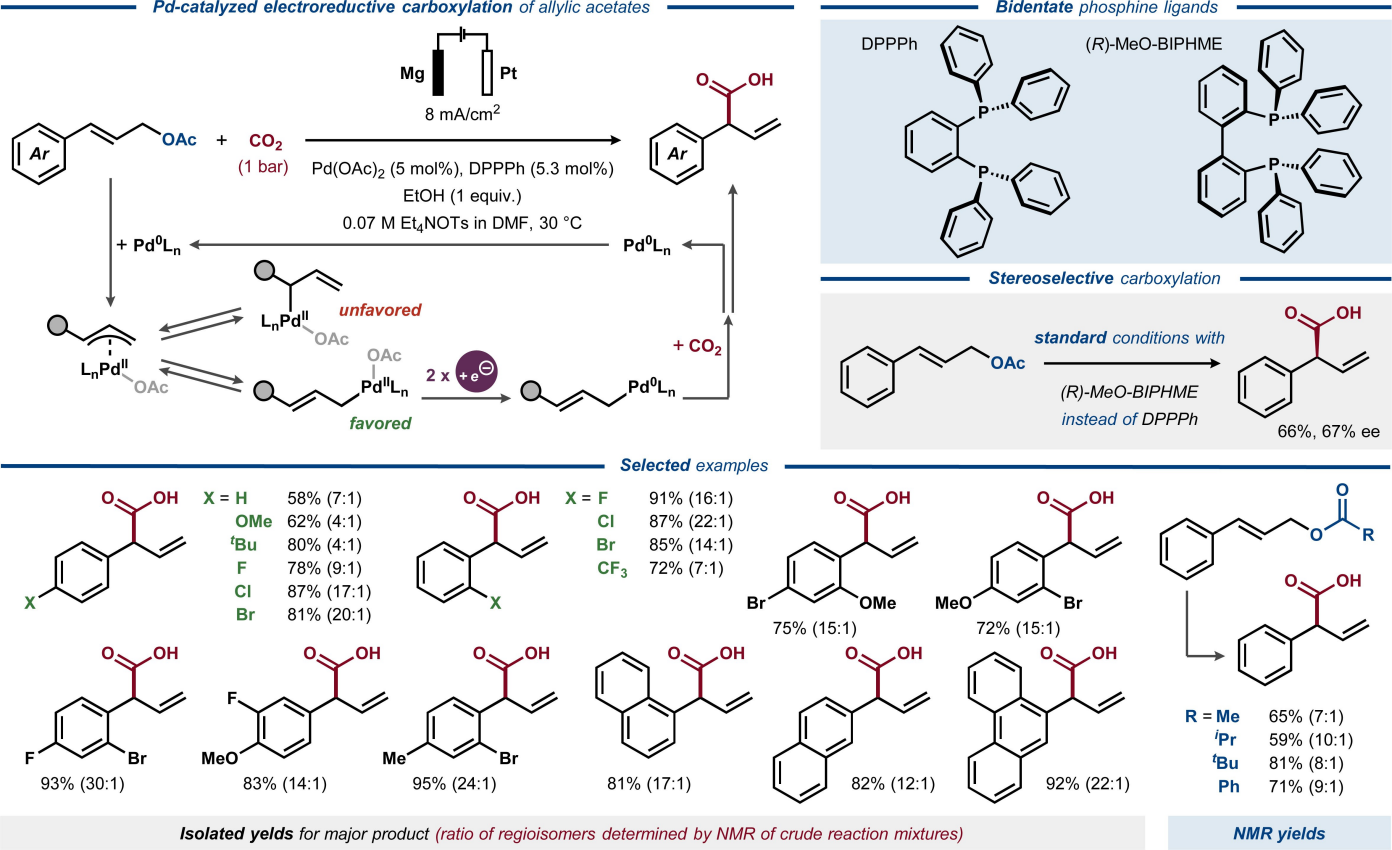
Electroreductive Pd‐catalyzed carboxylation of homostyrenyl acetates.

Under electroreductive conditions similar to those of Duñach,^[134]^ deoxygenative electrocarboxylation of aromatic homostyrenyl acetates was reported by Mei, using Pd catalysis (Figure 22).^[135]^ The use of ethanol as an additive was found pivotal for achieving high yields and regioselectivities. Contrary to the selectivity observed for aliphatic allylic acetates,^[134]^ the homostyrenyl acetate substrates preferentially provided the branched instead of the linear regioisomer of the desired products. Notably, the styrene substrates with electron‐withdrawing substituents provided better regioselectivity for branched products relative to the substrates with electron‐donating substituents, which is an opposite trend compared to the related non‐electrochemical Pd‐catalyzed allylation reactions.[^136^, ^137^] The use of a chiral phosphine bidentate ligand allowed an enantioselective electrocarboxylation reaction to occur with moderate *ee* (67%).

**Figure 23:**
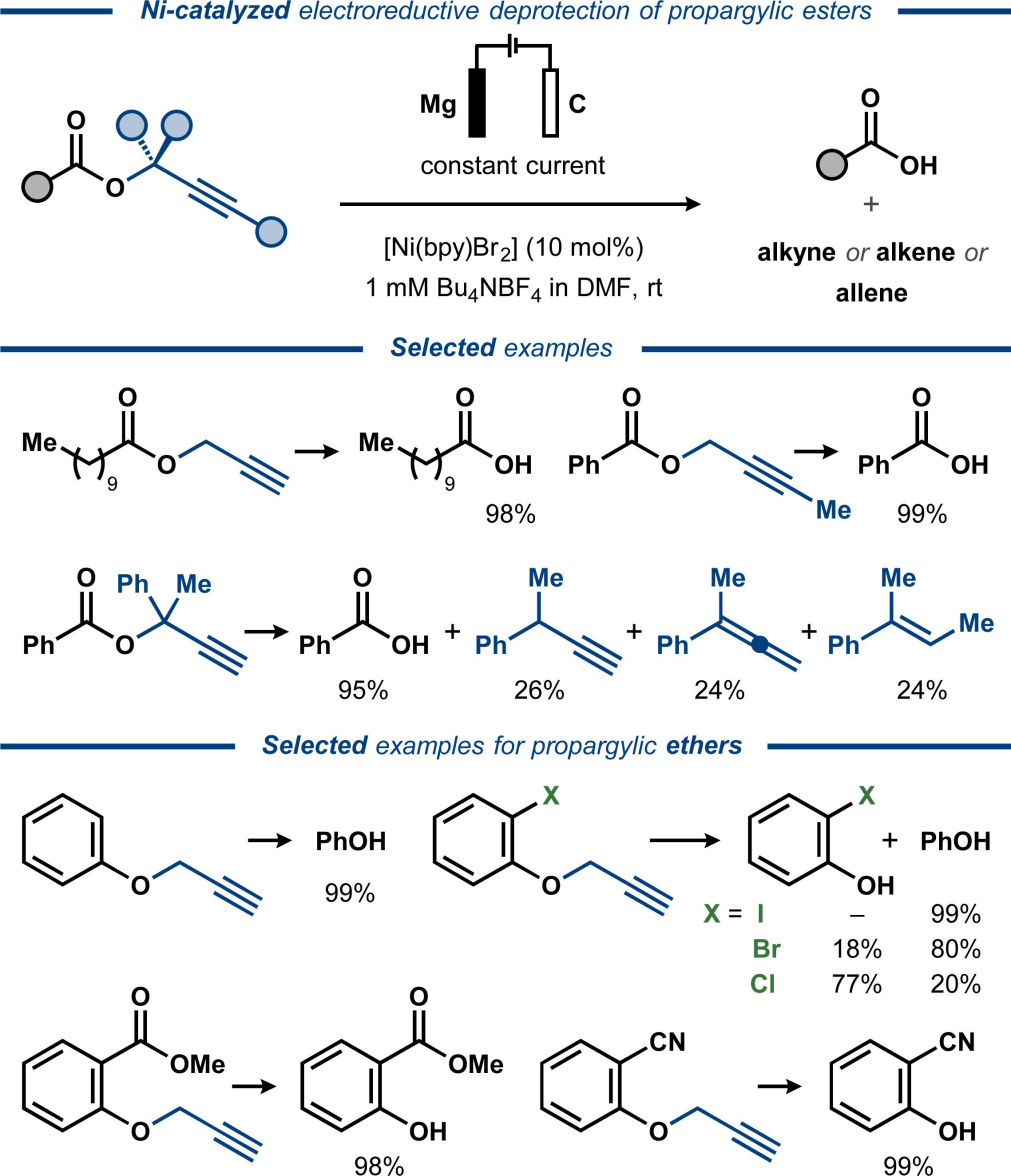
Electroreductive Ni‐catalyzed deprotection of propargylic esters and ethers.

**Figure 24:**
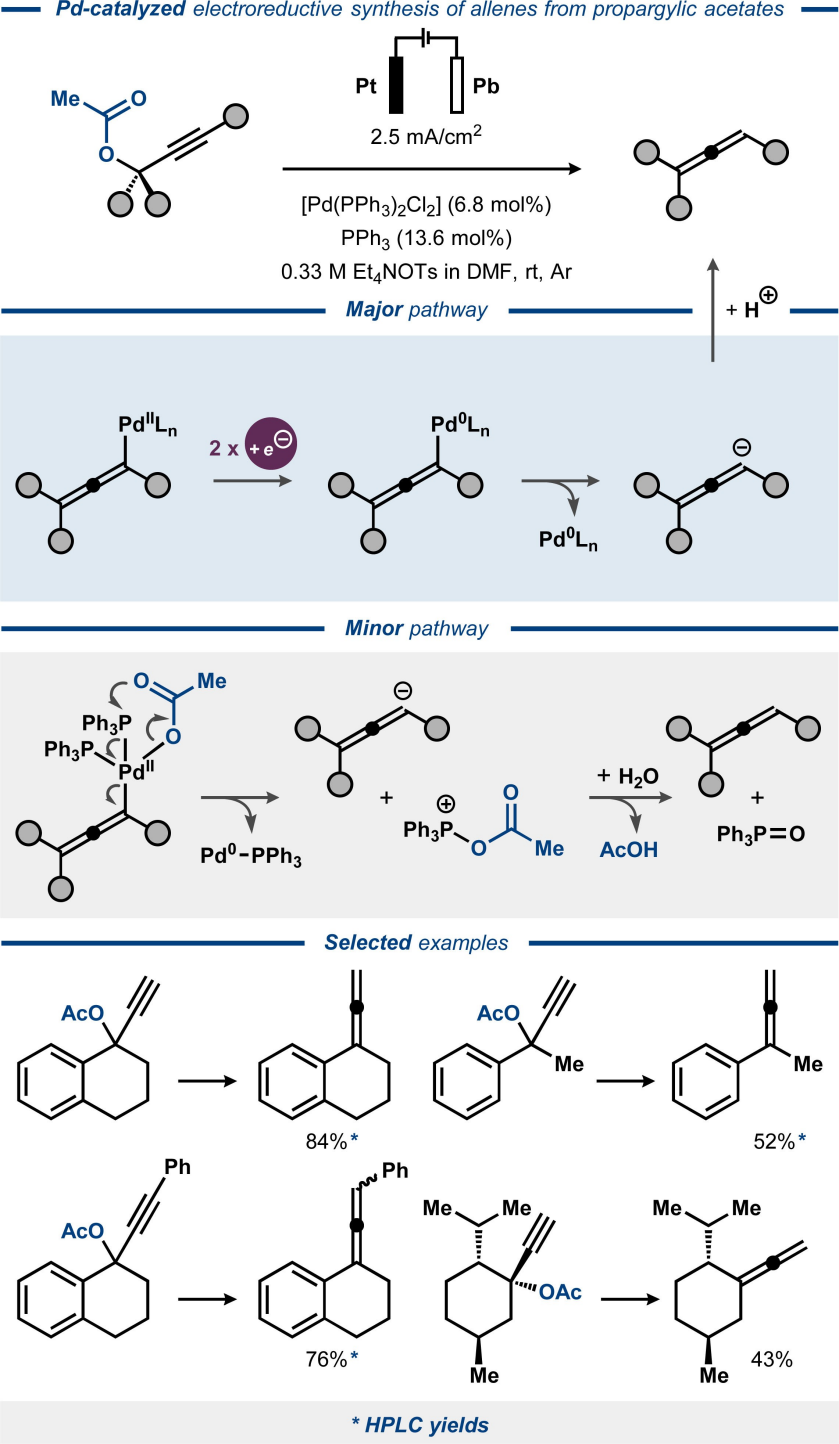
Electroreductive Pd‐catalyzed conversion of propargylic acetates to allenes.

In the 1990s, electroreductive cleavage of C(O)O−C bonds in propargylic esters was demonstrated with the aid of Ni and Pd catalysis by the groups of Duñach^[82]^ and Torii,^[138]^ respectively. For the Ni‐catalyzed approach, the propargylic moiety was exploited as a protecting group for carboxylic acids and alcohols (Figure 23).^[82]^ Here, electrolysis of several propargylic esters in an undivided cell with Mg anode and carbon fiber cathode in the presence of a Ni catalyst resulted in facile C−O bond cleavage and removal of the propargylic group. Presumably, the reaction proceeded through oxidative addition of an electrogenerated Ni^0^ catalyst to the C−O bond. The desired carboxylic acid products were liberated in near quantitative yields, while the propargylic functionality was unselectively converted into a mixture of alkyne, alkene and allene side‐products. In the related Pd‐catalyzed electrosynthetic system, electroreductive cleavage of propargylic acetates was instead used for preparative synthesis of substituted allenes, forming acetic acid as a by‐product.^[138]^ Here, the electrolysis was conducted in a divided cell, similar to the procedure for electroreductive cleavage of allylic acetates.^[128]^ A range of benzylic and aliphatic carbocyclic substrates were converted to the corresponding allenes in moderate to high yields (Figure 24). The reaction was proposed to proceed through oxidative addition of propargylic acetate to electrogenerated Pd^0^ catalyst, forming an allenyl Pd^II^ complex. Cathodic reduction of the latter results in the corresponding Pd^0^ species, which dissociates to regenerate the initial Pd^0^ catalyst and an allenyl anion that furnishes the desired allene product upon protonation.

Ni‐catalyzed deoxygenative cross‐coupling of allylic acetates and oxalates, as well as trifluoromethyl styrenes, with aryl halides was recently reported by Ni, Wang and co‐workers.^[139]^ Here, the C−O bond was indirectly cleaved via conjugate radical addition followed by β‐elimination to furnish the substituted products. Additionally, allylic alcohols could engage in the reaction under similar conditions. The use of cobalt catalysis has also been reported under electroreductive conditions, allowing facile deoxygenative dimerization of cinnamyl acetate to 1,6‐diphenyl‐1,5‐hexadiene.^[140]^

## 5 Electrochemical C−O Bond Activation in Carbonates and Carbazates

**Figure 25:**
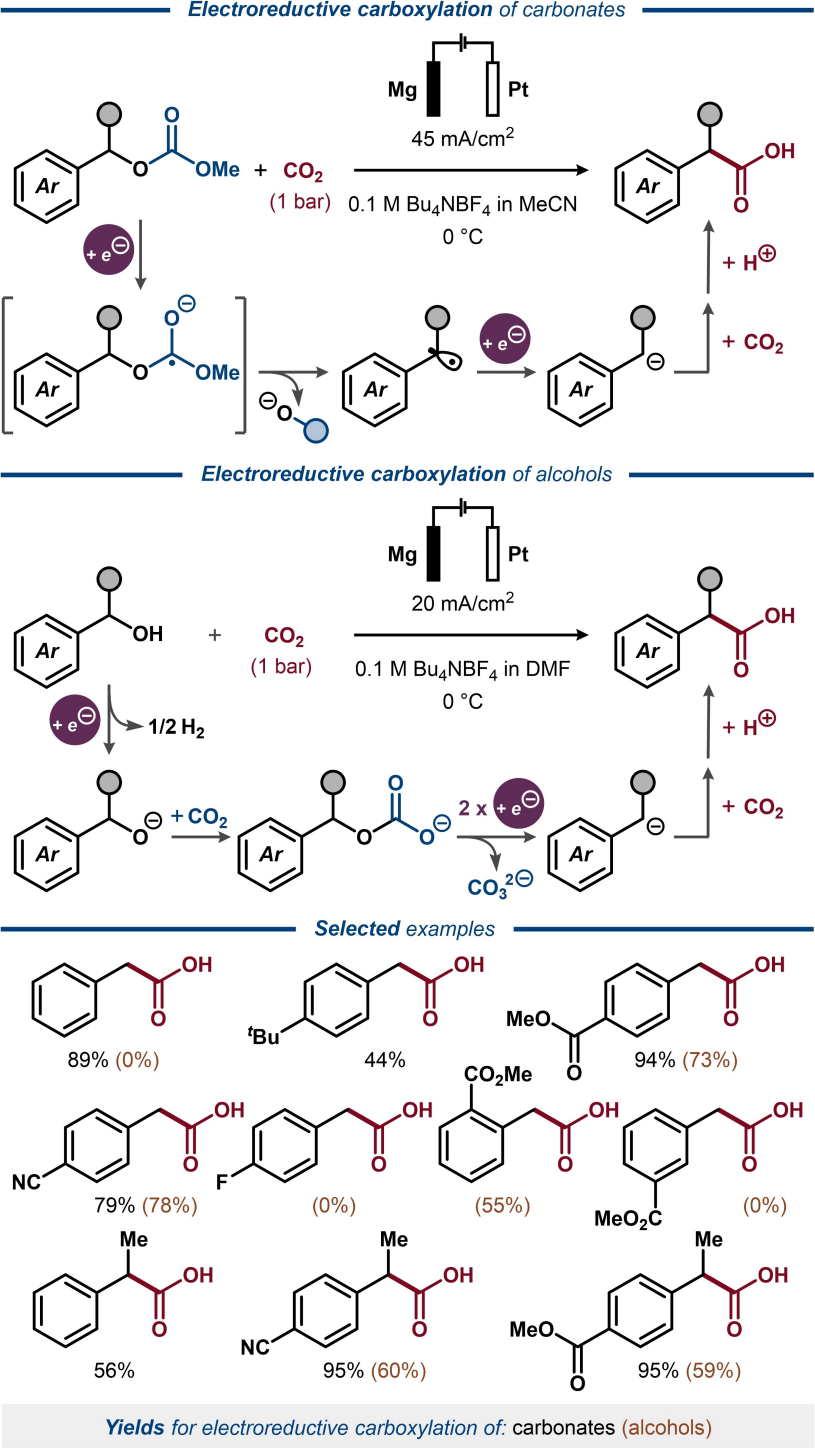
Electroreductive carboxylation of benzylic carbonates (top) and electroreductive carboxylation of benzylic alcohols through in situ conversion to benzylic carbonates (middle).

Carbonates,[^141^, ^142^, ^143^] carbamates,[^69^, ^142^, ^143^, ^144^, ^145^, ^146^] thiocarbamates^[141]^ and thioxocarbamates^[147]^ have been explored as electrochemically cleavable protecting groups for alcohols, amines and thiols, respectively, under non‐mediated electrolysis conditions. Although the electrochemical deprotection required highly oxidative or highly reductive potentials in most cases, some of the methods were applicable for selective removal of a specific carbonyl‐containing protecting group in the presence of other protecting groups with similar (electro)chemical stability.[^69^, ^143^, ^144^] An alternative type of reactivity featuring electroreductive C−O bond cleavage in carbonates was demonstrated under direct electrolysis conditions by Senboku.[^148^, ^149^] Here, methyl carbonates of benzylic alcohols were subjected to constant current electrolysis under a CO_2_ atmosphere (Figure 25, top).^[148]^ The carbonate substrate was proposed to undergo sequential two‐electron cathodic reduction concomitant with C(O)O−C bond cleavage, producing a benzylic carbanion, which reacts with CO_2_ to furnish an arylacetic acid product. A scope of methyl carbonates of primary and secondary benzylic alcohols formed the desired deoxygenative carboxylation products in good to excellent yields. A subsequent deoxygenative carboxylation protocol by Senboku detailed a related reaction where benzylic alcohols are transformed into arylacetic acids (Figure 25, middle). It was hypothesized that the benzylic alcohol in the presence of CO_2_ forms a monofunctionalized carbonate intermediate in situ,[^149^, ^150^] adopting a similar strategy to the transesterification‐promoted reductive deoxygenation of alcohols through in situ formation of *p*‐toluate esters and oxalates (Figures 16 and 17).[^109^, ^116^, ^117^] Under the optimized conditions, primary benzylic alcohols bearing strongly electron‐withdrawing substituents at the *para*‐position could be efficiently converted into the corresponding arylacetic acids, whereas moderately electron‐withdrawing groups proved inefficient. As expected, the reaction was less effective for secondary and tertiary benzylic alcohols proceeding through more destabilized benzylic carbanion intermediates. Despite a somewhat limited substrate scope, these methods represent a notable achievement among electroreductive carboxylation manifolds, most of which proceed through the cleavage of carbon−halogen bonds.[^151^, ^152^]

**Figure 26:**
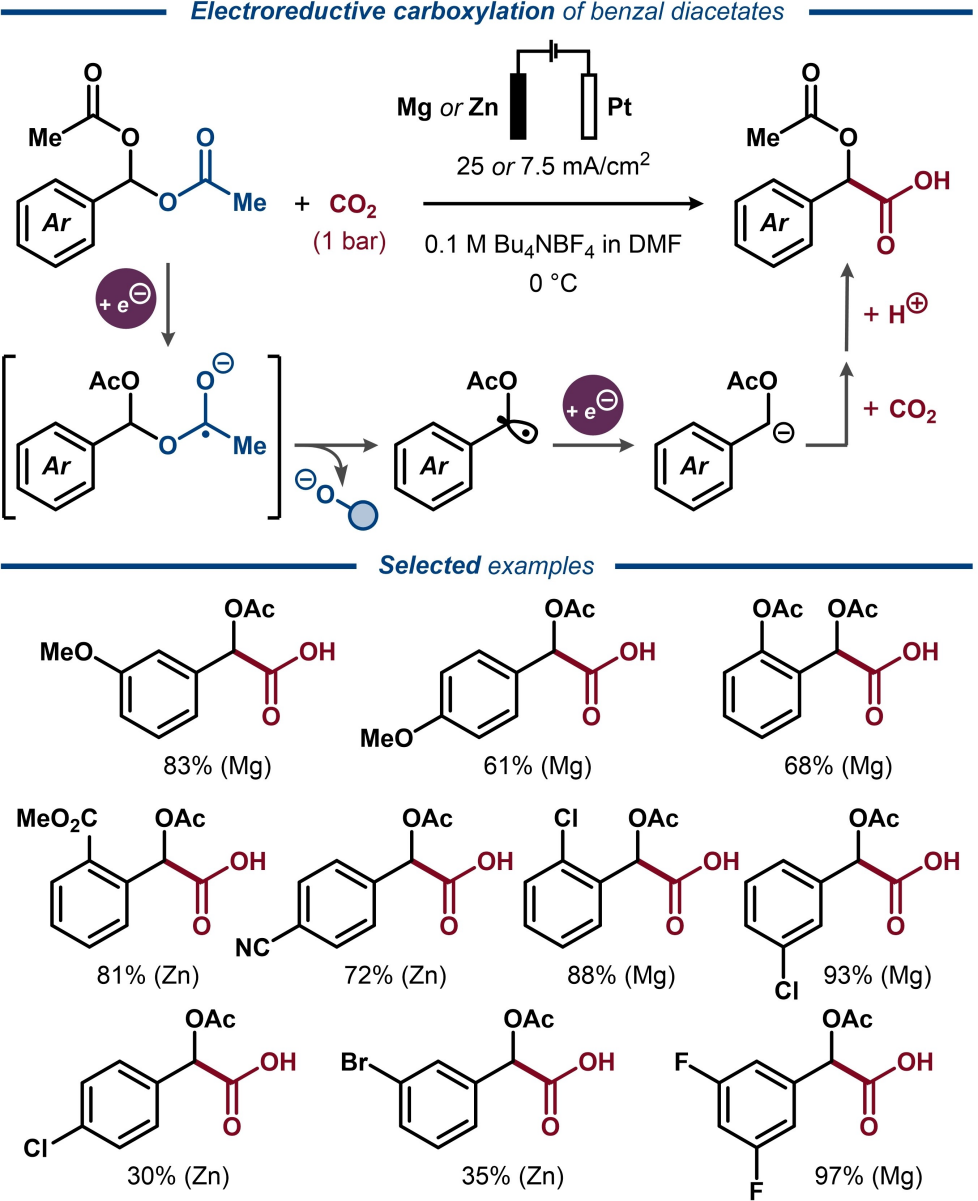
Electroreductive carboxylation of benzal diacetates.

Another approach for the synthesis of functionalized arylacetic acids was demonstrated by Senboku with benzal diacetates as the starting material (Figure 26).^[153]^ In this case, the reaction proceeds through cathodic one‐electron reduction of benzal diacetate to a transient anion‐radical, which undergoes a second one‐electron reduction, providing the key benzylic carbanion intermediate upon C(O)O−C bond cleavage. The carbanion then reacts with CO_2_ to deliver acetylated mandelic acid as the product. The reaction proceeded under similar conditions as for the electroreductive carboxylation of benzylic carbonates and alcohols (Figure 25),[^148^, ^149^] but displayed an improved functional group compatibility and benzal diacetates containing both electron‐donating and electron‐withdrawing substituents could be efficiently converted into the corresponding acetylated mandelic acids. For the substrates containing strongly electron‐withdrawing substituents, changing the anode material from Mg to Zn was shown to suppress over‐reduction of the acetylated mandelic acid products to the corresponding arylacetic acids.

**Figure 27:**
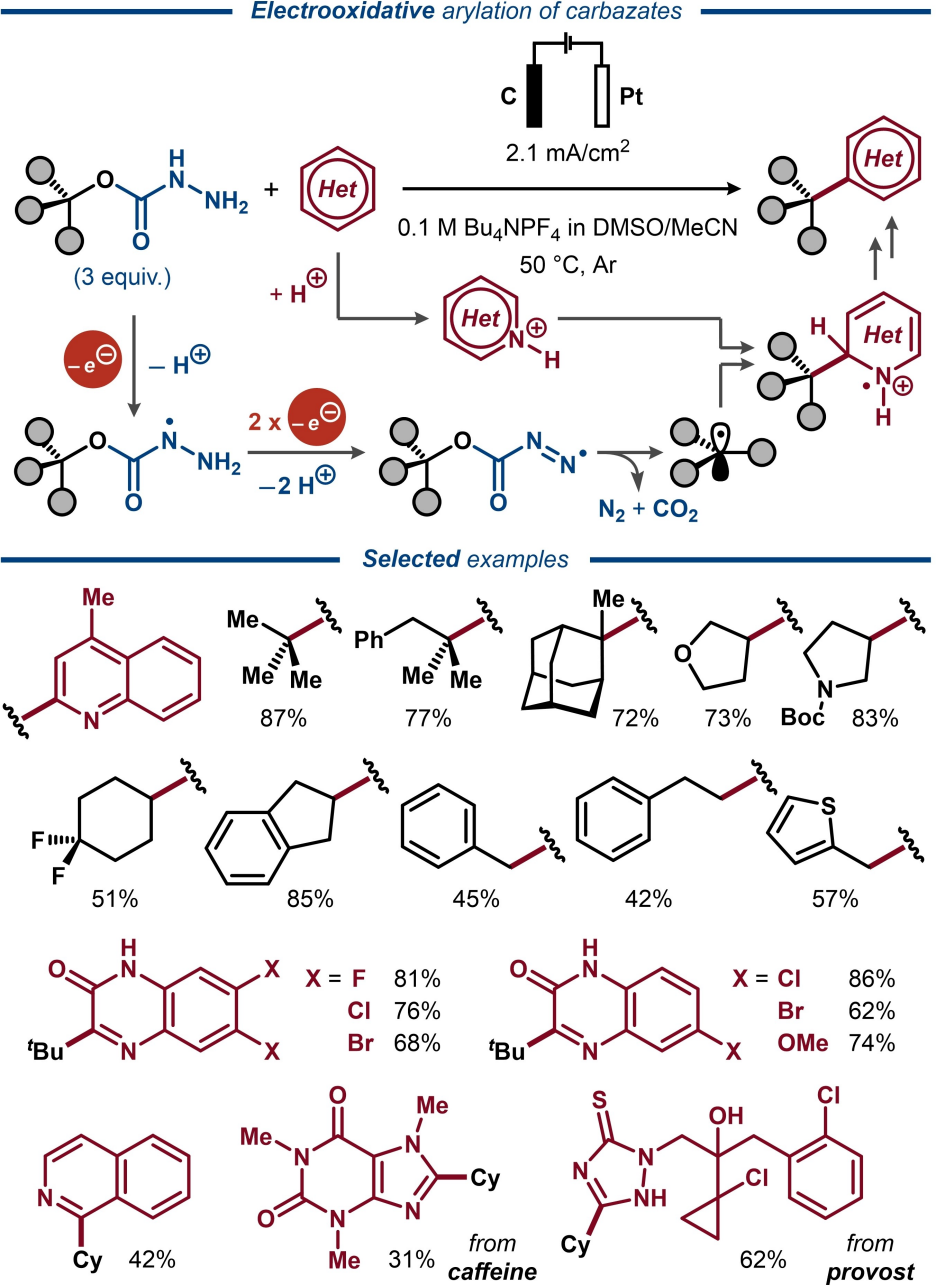
Electrooxidative heteroarylation of alkyl carbazates.

Recently, generation of alkyl radicals through direct electrooxidative cleavage of the C(O)O−C bond in alkyl carbazates was utilized in a Minisci‐type reaction by Wang and co‐workers (Figure 27).^[154]^ Here, the alkyl carbazates were proposed to undergo two consecutive one‐electron/one‐proton oxidation steps, furnishing a diazenecarboxylate ester, which undergoes a third one‐electron/one‐proton oxidation to furnish the key C‐radical intermediate upon elimination of N_2_ and CO_2_. The C‐radical engages in a C−C bond forming reaction with an N‐heterocyclic compound to form the Minisci‐type product upon one‐electron oxidation and rearomatization. The protocol was successfully applied for a wide range of alkyl carbazates prepared from tertiary, secondary and primary alcohols with a variety of heterocyclic radical acceptors, including pharmaceutical structures, such as caffeine and prothioconazole as well as substituted quinoxalinones.^[155]^

## 6 Electrochemical C−O Bond Activation in Sulfonate Esters

Conversion of alcohols into sulfonate esters is a classic synthetic strategy to weaken the C−O bond and facilitate nucleophilic substitution, transition metal‐catalyzed cross‐couplings, as well as electrochemical transformations. Single‐electron reduction of sulfonate esters results in anion‐radicals that can undergo either C−O or S−O bond cleavage. Electrochemical protocols for selective S−O bond cleavage have been used for deprotection of the parent alcohols,[^156^, ^157^, ^158^, ^159^, ^160^] while other protocols describe the formation of a mixture of alcohol and alkane products with a ratio that depends on both the reaction conditions and the nature of the substrates.[^161^, ^162^] Similarly, electrochemical studies of aryl and alkyl trifluoromethanesulfonate (triflate) esters revealed that the selectivity for C−O versus S−O bond cleavage is highly substrate‐dependent.[^163^, ^164^, ^165^]

**Figure 28:**
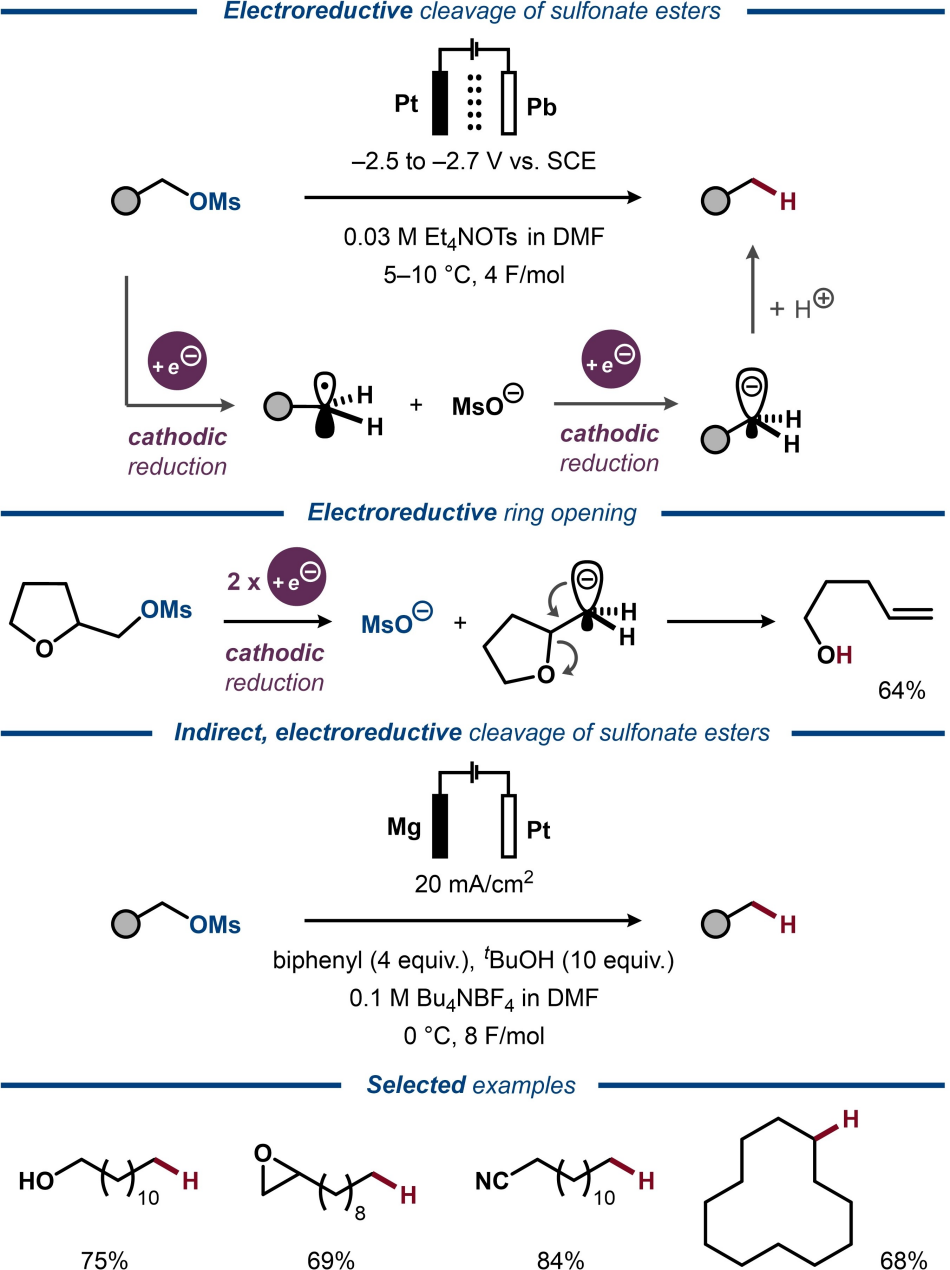
Deoxygenative cleavage of mesylates to form alkanes, top: in a divided cell; bottom: in an undivided cell in the presence of an electron‐transfer mediator and an H‐source.

In 1979, complete selectivity for C−O bond cleavage was reported by Shono for potentiostatic electroreduction of methanesulfonate (mesylate) esters into the corresponding alkanes in a divided cell (Figure 28, top).[^11^, ^12^] The procedure was demonstrated to tolerate various functional groups, including ester, nitrile, olefin, epoxide and hydroxy groups. The transformation was proposed to be initiated by single‐electron transfer to the mesylate to form the corresponding anion‐radical, which undergoes C−O bond cleavage to furnish a mesylate ion and a C‐radical. This species is reduced to a carbanion that is protonated to form the alkane product (Figure 28, middle). In 2007, Senboku reported that the use of biphenyl or other (poly)cyclic aromatic additives enabled conducting the reaction in an undivided cell (Figure 28, bottom).^[166]^ CV studies revealed that the aromatic additive was reduced at more anodic potentials compared to the mesylate ester and that the reduction current of the additive increased with the concentrations of the mesylate. Hence, the aromatic compound was proposed to act as a redox mediator in the system. Addition of a proton source was found beneficial for the yield of the deoxygenation products.

**Figure 29:**
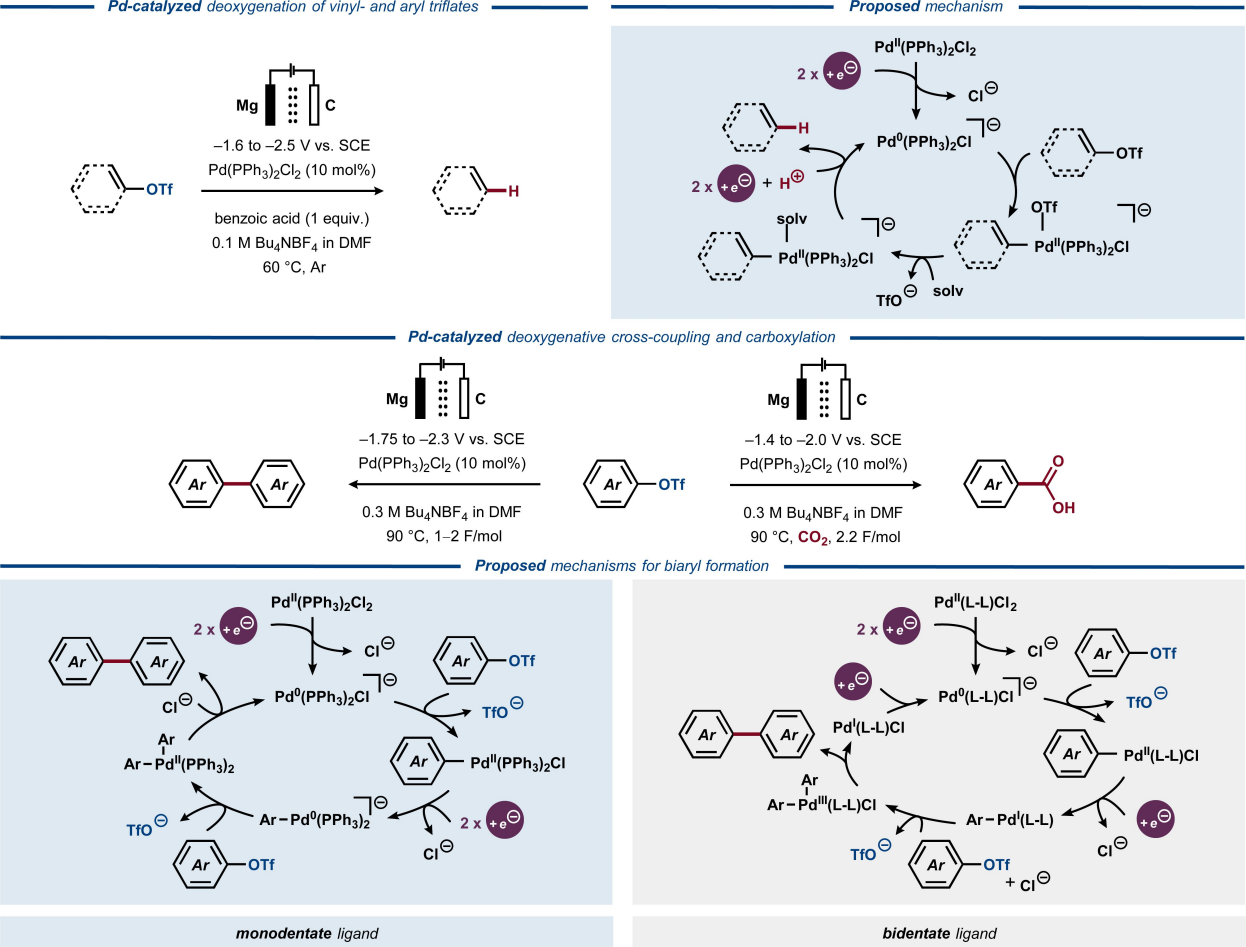
Electrochemically driven Pd‐catalyzed C−O bond activation and functionalization in aryl triflates.

Sulfonate esters are known to undergo oxidative addition to low‐valent late transition metal catalysts for subsequent cross‐coupling,[^167^, ^168^, ^169^] a feature that has been utilized in an electrosynthetic context. In 1995, Carelli and co‐workers reported on electroreduction of vinyl and aryl triflates to the corresponding alkenes and arenes (Figure 29, top).^[14]^ In the presence of stoichiometric amounts of benzoic acid and catalytic amounts of Pd(PPh_3_)_2_Cl_2_, several triflates were reductively deoxygenated with a functional group tolerance encompassing esters, ethers and aryl chlorides. The mechanistic studies suggested a catalytic cycle initiated by electrochemical reduction of the Pd^II^ precursor to Pd^0^ species, which undergoes oxidative addition with the triflate substrate. Subsequent protonation and electrochemical reduction of the catalyst furnishes the olefin or arene product and closes the catalytic cycle. Notably, the aryl/alkenyl–Pd^II^ complexes displayed reduction potentials that were more anodic compared to those of the parent triflates. Effectively, this enabled electrolysis to be carried out at reduction potentials that were up to 1 V more anodic compared to the non‐catalyzed direct electrolysis of triflate esters.

In the 1990s, Jutand and co‐workers used a related approach for electroreductive homocoupling of aryl triflates to biaryls (Figure 29, middle).^[170]^ While direct electroreduction of aryl triflates resulted in S−O bond cleavage to afford deprotected phenols, addition of a Pd catalyst altered the reactivity to selectively furnish biaryl products. The potential was set to match the reduction of the aryl–Pd^II^ complex, which was determined to occur at 50–370 mV more anodic potentials compared to the parent triflates. Under a CO_2_ atmosphere, biaryl formation was inhibited to favor formation of the corresponding aromatic carboxylic acids. Subsequently, the strategy was expanded to carboxylation of vinyl triflates to yield α,β‐unsaturated carboxylic acids.^[171]^

In 1997, the Jutand group disclosed a mechanistic study on reductive biaryl formation from aryl triflates.^[172]^ CV studies indicated that Pd‐catalyzed systems that employ monodentate phosphine ligands are activated by electrochemical reduction of the divalent Pd precursor to its zero‐valent state. The Pd^0^ secies undergoes oxidative addition to the aryl triflate, analogous to the initial stages of Carelli's mechanistic proposal.^[14]^ The resulting aryl–Pd^II^ complex undergoes a two‐electron reduction at the cathode to form a Pd^0^ complex, which undergoes oxidative addition to the second aryl triflate. Subsequent reductive elimination affords the biaryl product and regenerates Pd^0^ to close the catalytic cycle (Figure 29, bottom). In contrast, the addition of a bidentate ligand enables access to more unusual valence states of Pd, evoking both zero‐, mono‐, di‐ and trivalent oxidation states,^[172]^ resembling the rich redox behavior of Ni‐based catalysts.^[173]^ The Ni‐catalyzed reductive cross‐coupling required less elevated temperatures compared to the Pd‐catalyzed reaction, which was attributed to the higher rates of oxidative addition of the aryl triflate to Ni^0^ compared to its heavier congener.

In 1998, Jutand published an improved protocol for electroreductive Pd‐catalyzed carboxylation of aryl and vinyl triflates.^[174]^ A variety of carboxylic acids were formed in high yields and good functional group tolerance and the transformation was proposed to proceed through a similar mechanism to that of Pd‐catalyzed biaryl formation using monodentate phosphine ligands (Figure 29, bottom right). However, instead of a second oxidative addition to the aryl–Pd^0^ complex to furnish the biaryl product, the carboxylated product was proposed to form via attack on CO_2_ by the aryl anion claimed to reside in equilibrium with aryl–Pd^0^ complex. This proposal was supported by earlier studies of halogenated substrates by the same group.^[175]^

**Figure 30:**
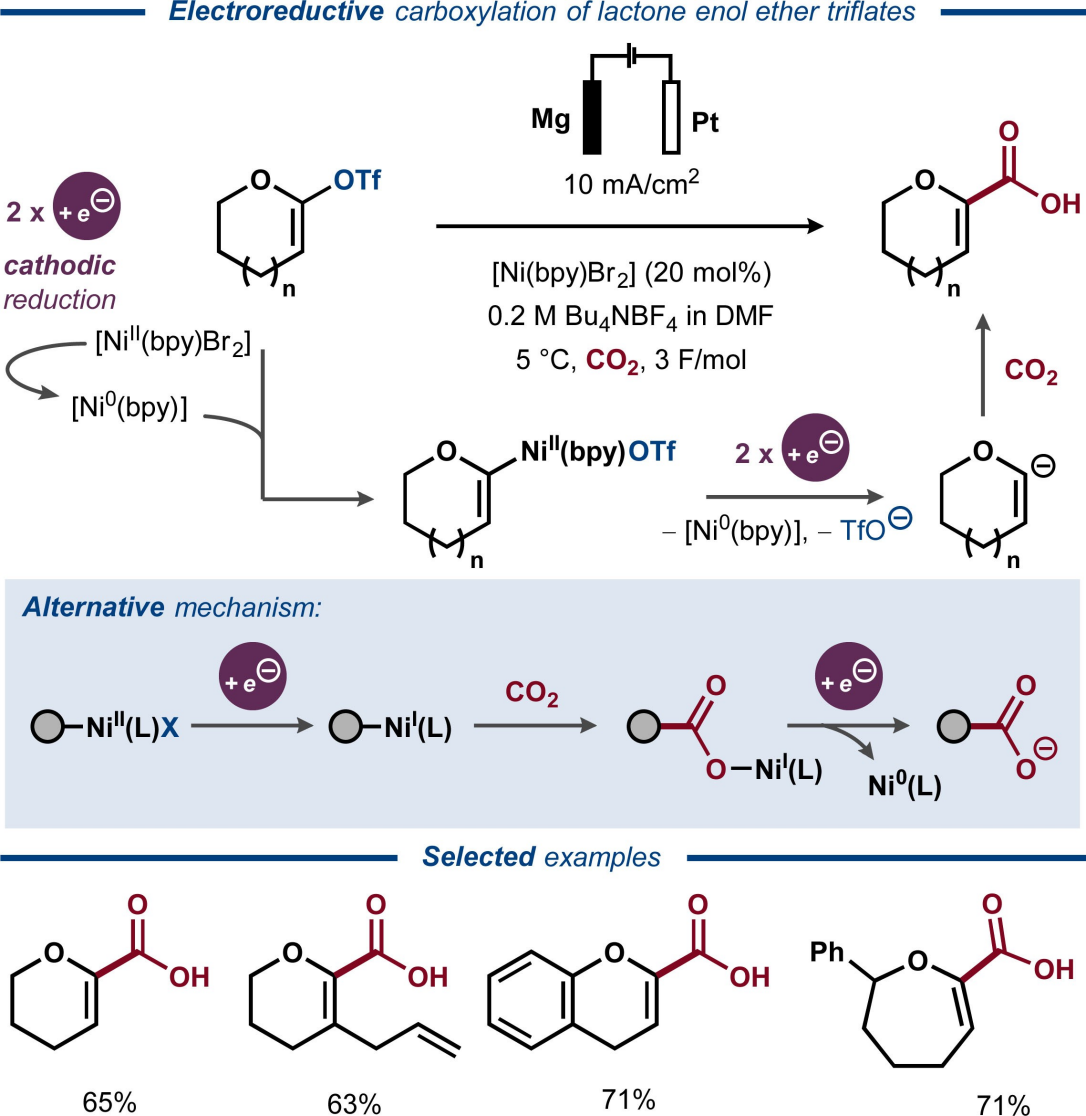
Electroreductive Ni‐catalyzed carboxylation of enol ether triflates.

In 2002, Senboku, Tokuda and co‐workers disclosed a protocol for electrochemical carboxylation of enol triflates using Ni‐catalysis (Figure 30).^[176]^ Several cyclic α,β‐unsaturated carboxylic acids were formed with full selectivity for C−O bond cleavage, which stands in contrast to the highly substrate‐dependent chemoselectivity for C−O versus S−O bond cleavage observed in previous studies under similar conditions.[^163^, ^164^, ^165^] The proposed mechanism is initiated by oxidative addition of the enol triflate to the Ni^0^ catalyst, followed by a two‐electron reduction to furnish a C(sp^2^)‐centered anion that reacts with CO_2_ to afford the carboxylate product. This proposal stands in contrast to more recent literature for analogous Ni‐catalyzed reductive carboxylation of aryl halides and pseudohalides, including carboxylic and sulfonate esters.[^177^, ^178^] Here, the product of oxidative addition to the Ni^II^ complex is instead proposed to undergo single‐electron reduction to the corresponding aryl–Ni^I^ species, after which CO_2_ inserts into the Ni−C bond to form the corresponding Ni–carboxylate that dissociates the desired product upon a second single‐electron transfer.

**Figure 31:**
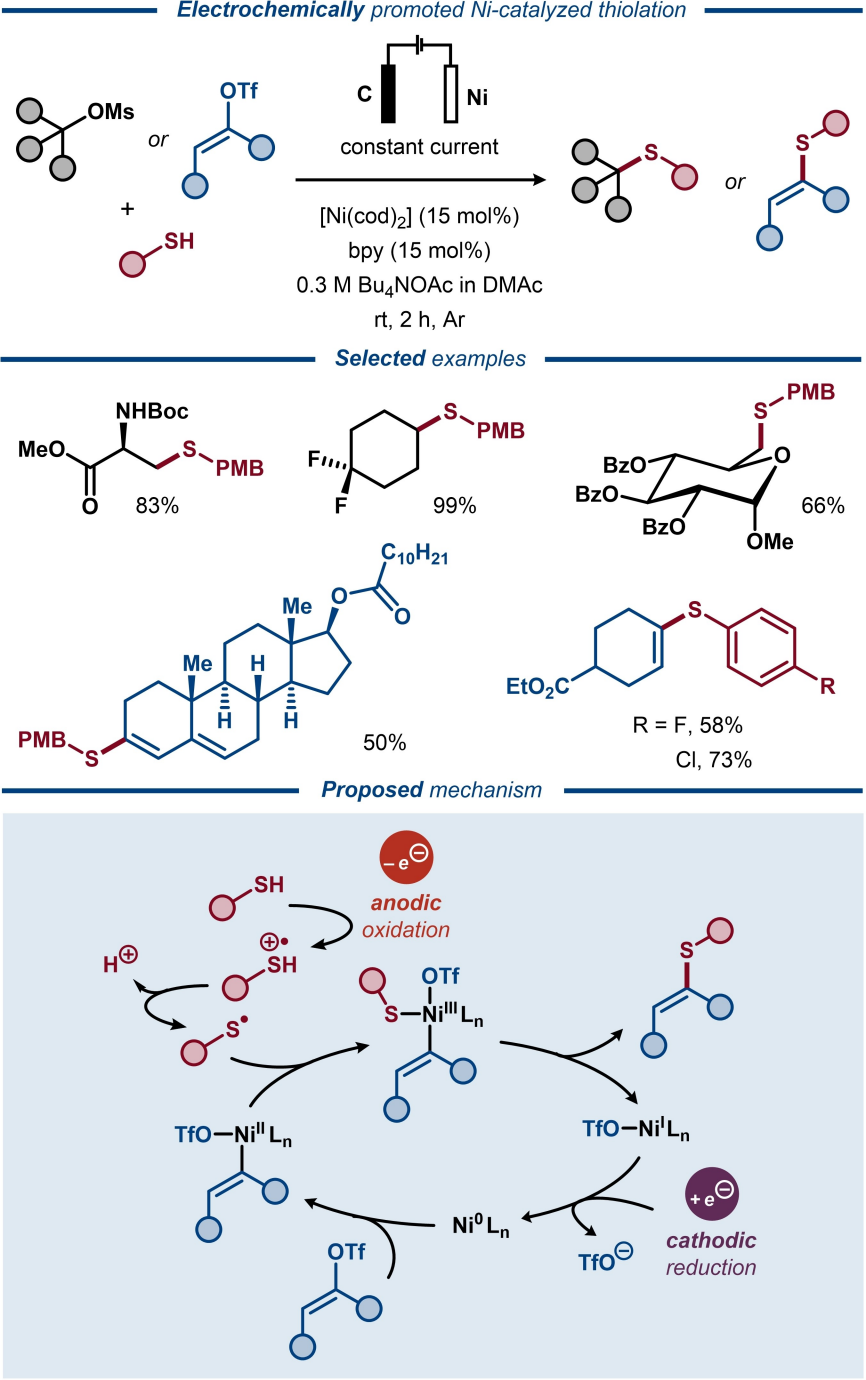
Electroreductive Ni‐catalyzed deoxygenative cross‐coupling of vinyl triflates and thiols.

Recently, Wang, Wang and co‐workers disclosed a paired Ni‐catalyzed electrochemical protocol for deoxygenative cross‐coupling of vinyl triflates or alkyl mesylates with thiols to afford thioethers (Figure 31).^[179]^ A wide range of thioethers were obtained in up to quantitative yields with good functional group tolerance. The protocol was amenable to gram scale synthesis and the coupling of vinyl triflates could be carried out in a one‐pot fashion starting from the carbonyl compound and triflic anhydride. The major mechanistic pathway was proposed to commence through oxidative addition of the sulfonate ester to the Ni^0^ catalyst. Upon reaction of the resulting Ni^II^ complex with a thiyl radical, formed upon anodic oxidation of the thiol, a trivalent Ni^III^ species is formed. This species undergoes reductive elimination to afford the thioether product and a Ni^I^ complex, which is reduced to the Ni^0^ state at the cathode, thereby closing the catalytic cycle.

## 7 Electrochemical C−O Bond Activation in Phosphorous‐Based Alcohol Derivatives

**Figure 32:**
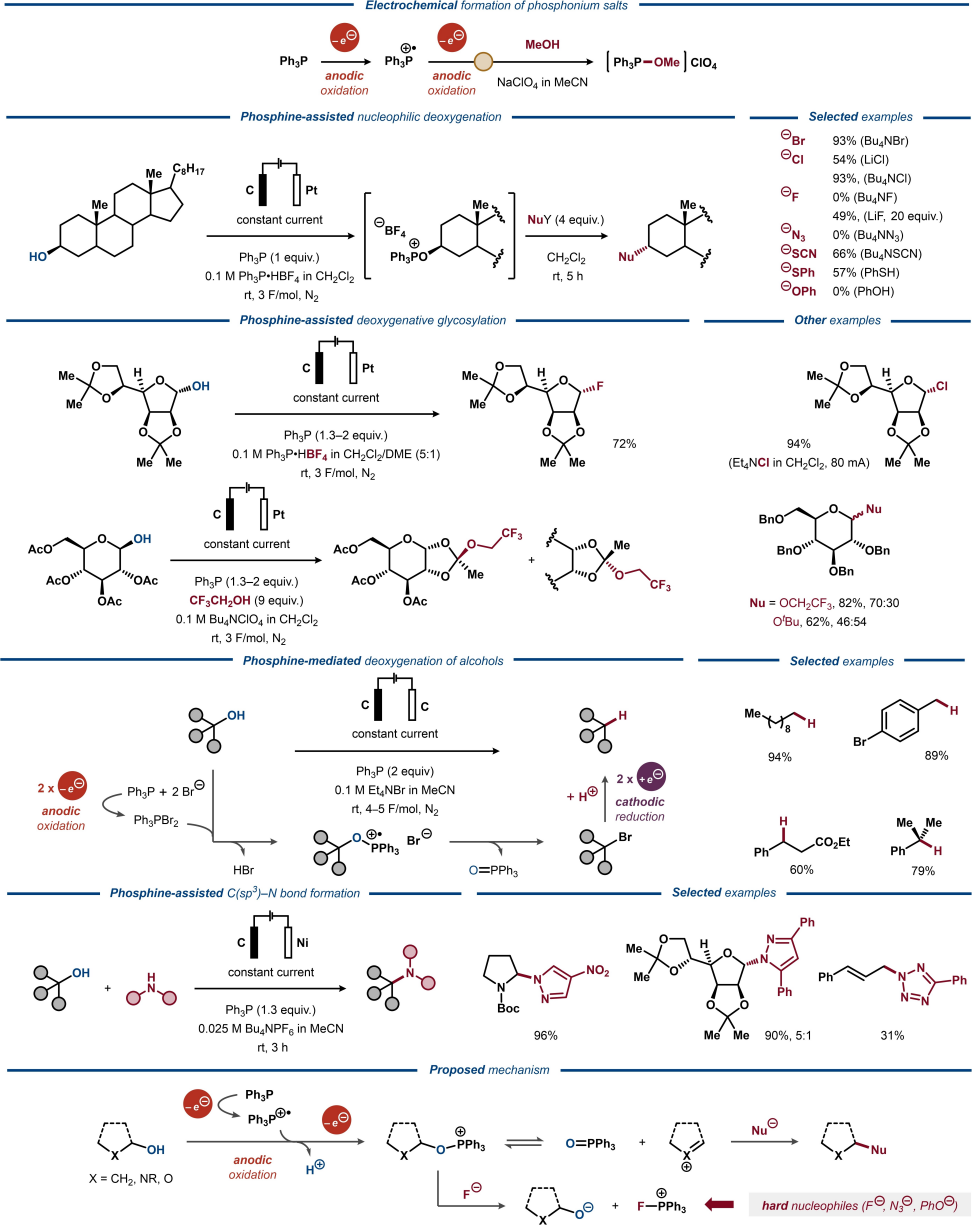
Phosphine‐promoted deoxygenative activation of alcohols. Top: Preparation of alkoxy phosphonium salts via anodic oxidation. Second from top: Nucleophilic substitution of alkoxy phosphonium salts. Third from top: Deoxyfluorination of alkoxy phosphonium salts. Fourth from top: Dehydroxylative synthesis of alkanes from alcohols via alkoxy phosphonium salts. Bottom: Dehydroxylative C−N formation via alkoxy phosphonium salts.

Trivalent phosphorous reagents and catalysts have long been known to facilitate cleavage of C−O bonds in both two‐ and one‐electron processes, driven by the concomitant formation of phosphine oxide. This synthetic strategy has been utilized in several classical transformations, including the Wittig olefination, the Appel reaction and the Mitsunobu reaction, as well as more recent protocols under chemical and photochemical conditions.[^180^, ^181^, ^182^, ^183^, ^184^] Phosphines have also been successfully used for deoxygenative transformations of carboxylic acids and alcohols under electrochemical conditions.[^185^, ^186^, ^187^, ^188^] In the 1980s, Ohmori and co‐workers realized that PPh_3_ could be anodically oxidized and reacted with alcohols and thiols to form isolable phosphonium salts of the same kind as the postulated intermediates in the Appel and Mitsunobu reactions (Figure 32, top).^[189]^ As expected from the reactivity in the parent reactions, these electrochemically generated alkoxy phosphonium salts were successfully applied as alkylating agents for subsequent functionalization of imidazoles, thiophenols and carboxylic acids. The chemoselectivity of the alkylation reactions was investigated in more detail in a subsequent study (Figure 32, second from top).^[190]^ Softer nucleophiles, such as chloride, bromide, thiocyanate and thiol, were prone to attack the alkoxy phosphonium salt at the alkoxy carbon through S_N_2 mechanism. In contrast, harder nucleophiles, including fluoride, azide and phenol, did not furnish the substitution products, but instead regenerated the alcohol. This reactivity was rationalized as the result of a preferential attack by harder nucleophiles on the phosphorous center to form a phosphorane, with subsequent release of the alcohol. By tuning the reaction conditions, a selectivity switch was enabled using fluoride as nucleophile. In 1996, an improved PPh_3_‐assisted one‐pot protocol for deoxyfluorination of alcohols was developed, where the C(sp^3^)−F bond was formed via thermal decomposition of in situ formed alkoxy triphenylphosphonium salts with the BF_4_ counterion of a protic supporting electrolyte (PPh_3_⋅HBF_4_) serving as the fluoride source.^[191]^ The procedure was exemplified for a limited scope of primary and secondary alcohols, including cholestanol. A related protocol for anomeric substitution of carbohydrates with halides was developed several years later (Figure 32, third from top).^[192]^ Using this procedure, the glycosyl fluoride from diacetone α‐D‐mannofuranose was obtained in 72 % yield. Only the α‐anomer of the product was observed and it was hypothesized that the alkoxy phosphonium salt decomposes into an oxonium ion that reacts with the fluoride through an S_N_1 pathway from the less hindered α‐face. The same mechanism was proposed for the formation of the corresponding glycosyl chloride product, for which the aprotic salts Bu_4_NClO_4_ or Et_4_NCl were used as supporting electrolytes. Attempts to deoxyfluorinate glycopyranose derivatives under similar conditions resulted in low yields or recovered starting material, whereas the corresponding chlorination proceeded with moderate to good yields. O‐Glycosylation with protected furanose and pyranose substrates was also assessed for a selection of alcohols. Similar to earlier studies,^[190]^ it was found that alcohols with low steric bulk preferentially attack the phosphorous center to form new alkoxy phosphonium ions with O‐glycosylated products in only minor quantities. In contrast, the tertiary and perfluorinated alcohols produced O‐glycosylated products in high yields. The use of benzyl‐protected glucopyranosides was crucial, whereas acetyl‐protected derivatives afforded 1,2‐orthoacetates, likely via intramolecular addition of the neighboring acetyl group to the oxonium intermediate.

In addition to the nucleophilic substitution of the electrochemically generated alkoxy phosphonium species, Ohmori developed an electrochemical one‐pot procedure for cathodic reduction to the corresponding alkanes (Figure 32, fourth from top).^[193]^ While the one‐pot procedure resulted in the elimination product along with the desired alkane for certain secondary and tertiary alcohols, the selectivity was improved by tuning the electronic nature of the phosphine.

Using the alkoxy phosphonium strategy, Tian and Wang developed an electrochemical procedure for dehydroxylative C(sp^3^)−N bond formation in 2019 (Figure 32, bottom).^[194]^ The electrochemically formed alkoxy phosphonium salts were subjected to azoles or amides to form a wide range of C−N coupled products. Recent DFT calculations suggested that the initial one‐electron oxidation of the phosphine results in a cation‐radical that is attacked by the alcohol, followed by loss of a proton to form a P‐radical species.^[195]^ The latter is transformed into the alkoxy phosphonium ion upon the second one‐electron oxidation (Figure 32, bottom). In the absence of stabilizing heteroatoms, the alkoxy phosphonium salts can be directly attacked at the alkoxy carbon by nucleophiles, such as chloride and bromide ions, through an S_N_2 pathway. In the presence of electron‐deficient arenes as coupling partners, the same authors recently disclosed a related paired electrolysis protocol for C(sp^3^)−C(sp^2^) bond formation via radical–radical coupling.^[196]^

**Figure 33:**
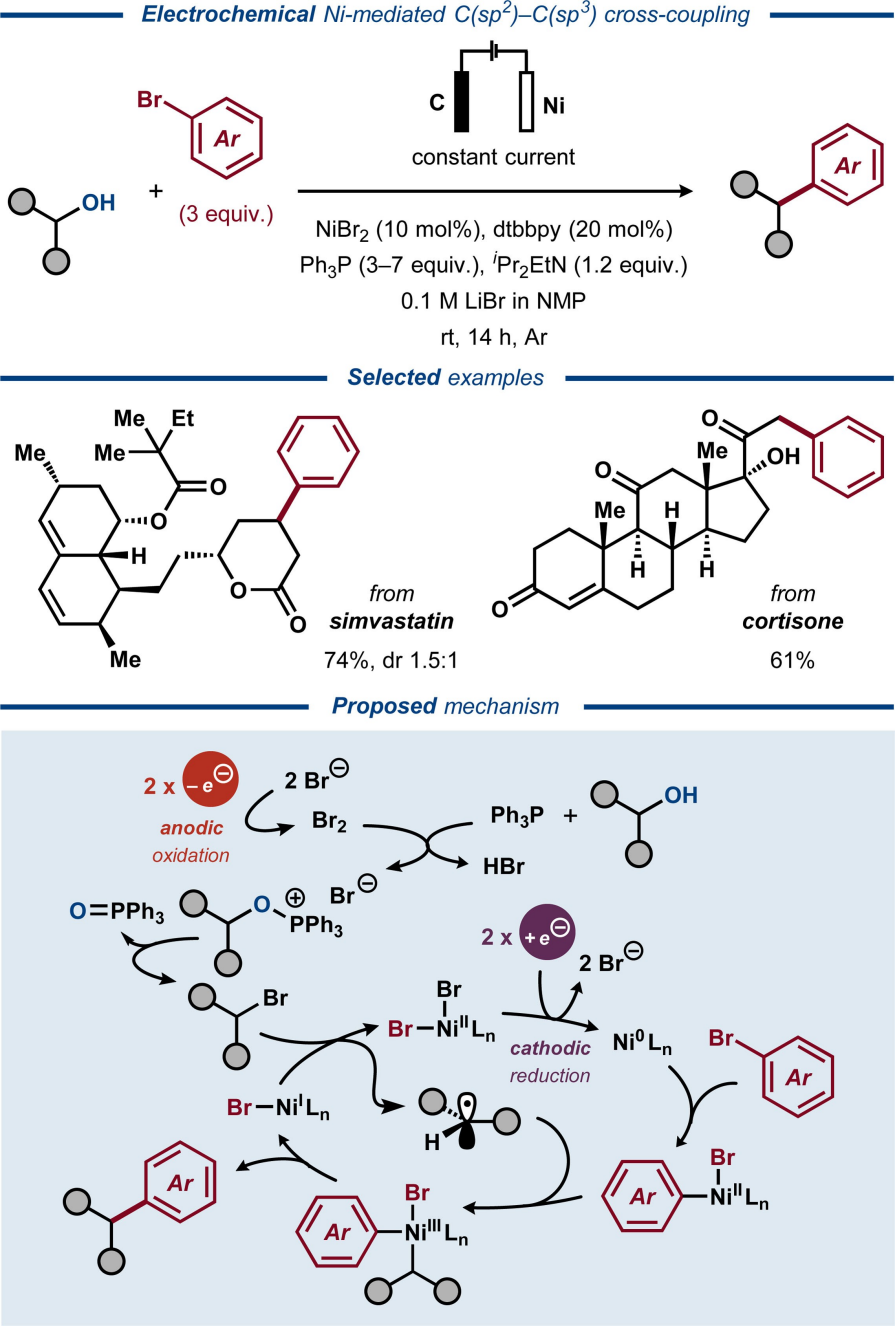
PPh_3_‐assisted and Ni‐catalyzed dehydroxylative C(sp^3^)−C(sp^2^) bond formation.

Recently, Li and co‐workers disclosed an electrochemically driven protocol for deoxygenative C(sp^2^)−C(sp^3^) cross‐coupling of alcohols and aryl halides in which the alkoxy phosphonium chemistry was combined with Ni catalysis (Figure 33).^[197]^ By merging the anodic generation of alkoxy triphenylphosphonium salts with Ni‐catalyzed cathodic cross‐coupling, this protocol showcased a paired electrosynthetic method for the construction of C−C bonds. Primary and secondary alcohols were successfully used as substrates in the transformation that tolerated a variety of functional groups and allowed for arylation of structurally complex natural products and pharmaceutically relevant motifs. The concentration of LiBr was found pivotal for the yield of the reaction and mechanistic studies indicated that the in situ formed alkoxyphosphonium ion is substituted by a bromide ion to afford the corresponding alkyl bromide in an electrochemically induced Appel‐type reaction. Thereafter, the alkyl bromide undergoes electrochemically driven Ni‐catalyzed cross‐electrophile coupling in a radical pathway, well‐established for this compound class.

**Figure 34:**
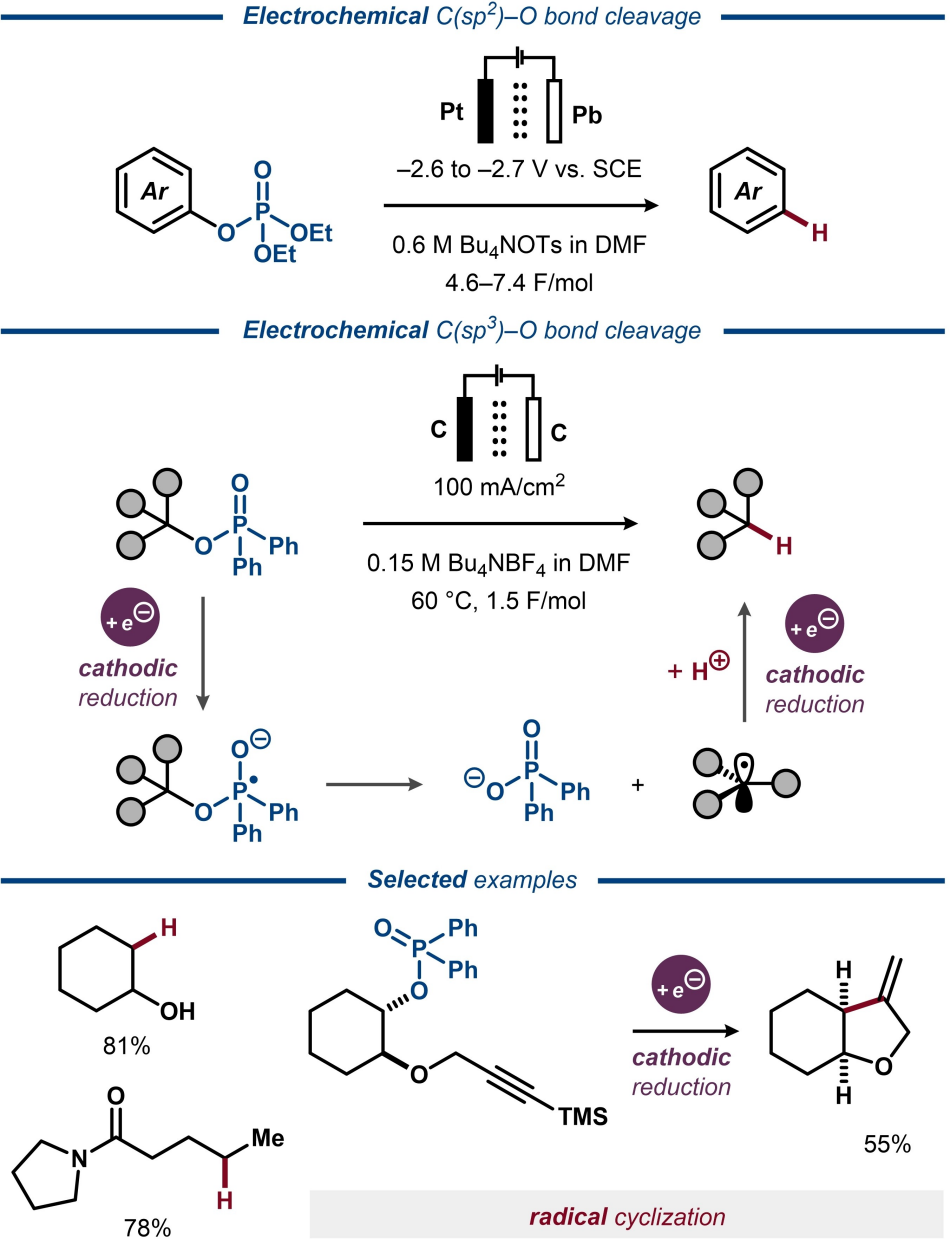
Deoxygenative alkane formation from phosphate and phosphinate esters.

While pentavalent phosphorous reagents are less frequently utilized in organic synthesis compared to their trivalent counterparts,^[198]^ deoxygenative transformations of phosphate and phosphinate esters have been reported under both chemical and electrosynthetic conditions. In 1979, Shono and co‐workers demonstrated that C(sp^2^)−O bonds were selectively cleaved over C(sp^3^)−O bonds in aryldiethylphosphates to furnish a variety of deoxygenated phenol derivatives (Figure 34, top).^[12]^ In addition to cleavage of the C−O bond, unsaturated substituents, such as allyl and propenyl groups, were reduced under the applied conditions. Due to the observed inertness of the C−O bonds in analogous aliphatic phosphate esters, the aromatic ring was proposed to play an active role in the initial electron transfer. Deuterium labeling experiments indicated that the hydrogen in the product originates in part from the traces of water in the reaction mixture, whereas the DMF solvent is an unlikely hydrogen source. In the 1990s, electroanalytical studies of C(sp^2^)−O bond cleavage of similar aryl phosphate esters to the corresponding hydrocarbons were carried out by Budnikova and co‐workers.[^199^, ^200^] The cleavage was postulated to proceed via two initial single‐electron transfers to the phosphate ester to afford a phosphate anion and a carbanion, followed by protonation of the latter to afford the hydrocarbon product.[^41^, ^201^, ^202^, ^203^] Recently, Tian and Wang and co‐workers demonstrated that aliphatic phosphate esters can undergo electrochemical deoxygenative C(sp^3^)−C(sp^3^) couplings with aldehydes and ketones to furnish alcohol products.^[204]^ Mechanistically, it was proposed that dissociative electron transfer to the phosphate ester furnishes an aliphatic radical that undergoes a second electron transfer to form the carbanion. This species would attack the carbonyl carbon of the coupling partner in a Barbier‐like sequence and form the alcohol upon protonation. Alternatively, the intermediate radical can undergo radical–radical coupling with a ketyl anion‐radical, formed by cathodic reduction of the carbonyl compound.

In a similar vein to Shono,^[12]^ Markó and Lam demonstrated in 2011 that primary, secondary and tertiary alcohols functionalized as diarylphosphinates undergo deoxygenation under electrochemical conditions to afford the corresponding hydrocarbons (Figure 34, bottom).^[13]^ The protocol was compatible with several functional groups, such as ketones, esters, amides, alkenes, silyl ethers and hydroxy groups. The authors proposed a radical mechanism of an EC‐type, where initial reduction of the diphenylphosphinate ester results in the corresponding anion‐radical, which undergoes fragmentation to diphenylphosphonic acid and a C‐radical, leading to the alkane product.

**Figure 35:**
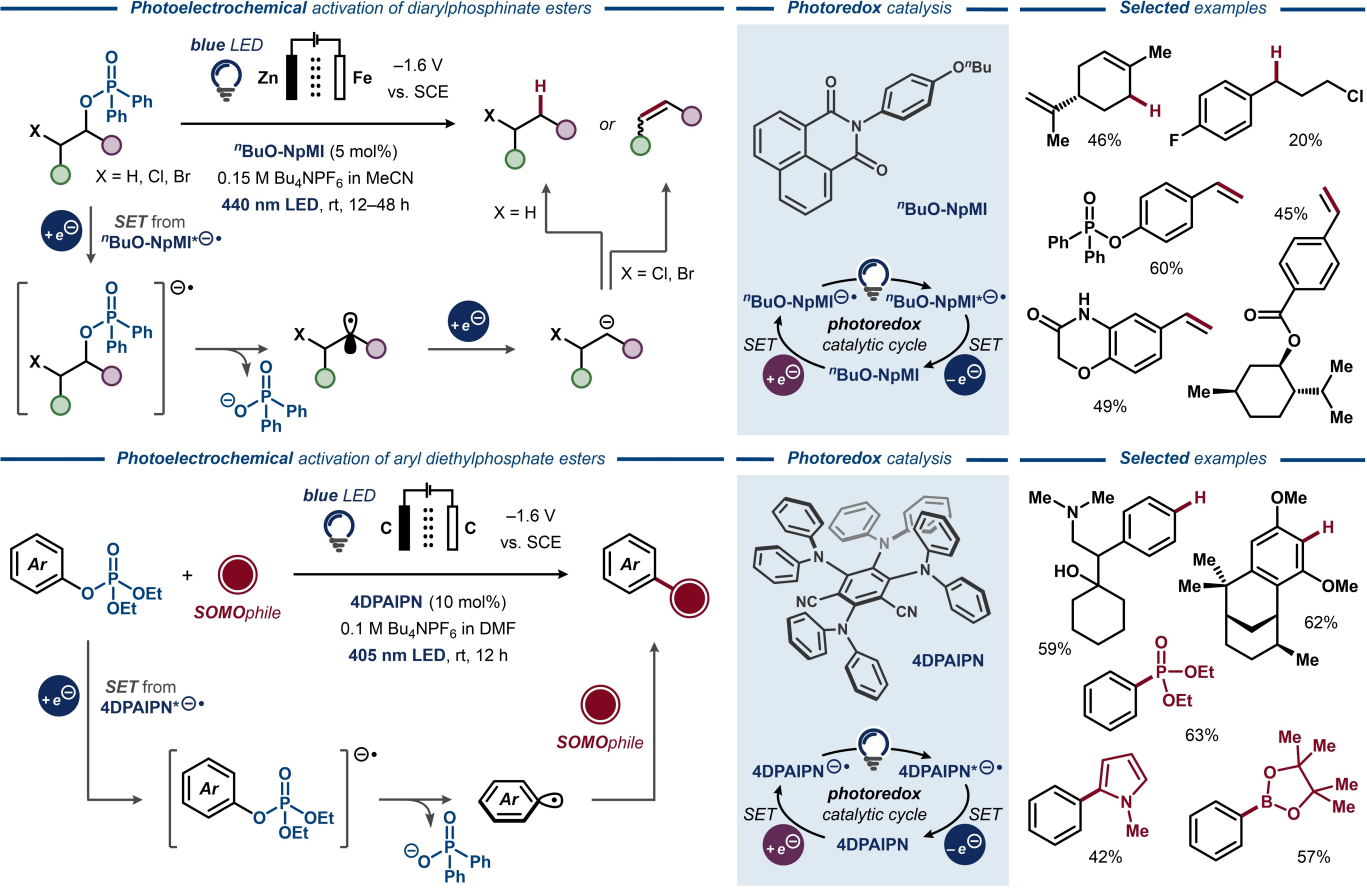
Photoelectrochemical deoxygenative transformations of phosphate and phosphinate esters.

Recently, photoelectrochemical protocols for C−O bond cleavage in phosphate and phosphinate esters were reported. In 2021, König and Barham disclosed a protocol for reductive C−O bond cleavage of diarylphosphinate esters (Figure 35, top).^[205]^ A combination of cathodic reduction and photoexcitation of the photocatalyst provided a catalytic species with dramatically altered redox potentials (−2.8 V vs SCE) compared to its reduced non‐excited state (−1.3 V vs SCE). Thus, the transformation was successfully achieved at a considerably more anodic potential (−1.6 V vs SCE) compared to what would be required for direct electroreduction of the phosphinate esters (−2.2 to −2.6 V vs SCE). Mechanistically, the transformation was proposed to proceed via initial formation of an anion‐radical upon quenching of the excited state of the reduced photocatalyst by the substrate. The anion‐radical undergoes C−O bond cleavage to form a C‐centered alkyl radical that is reduced to the corresponding carbanion, generating the alkane upon protonation. Compared to the direct electrochemical reduction protocol by Markó and Lam,^[13]^ the mild reaction conditions were found to tolerate reducible groups, such as aryl chlorides and bromides.

With similar operating principles to those proposed by König and Barham, Wickens and co‐workers disclosed a photoelectrochemical protocol for reductive cleavage of aryl phosphate esters in 2021 (Figure 35, bottom).^[206]^ Here, C(sp^2^)−O bonds in phenol‐derived phosphate esters were efficiently cleaved to furnish the corresponding arene products. The transformation was presumed to proceed via an intermediate C(sp^2^)‐centered radical that was successfully demonstrated to couple with phosphite esters, diboron compounds and *N*‐methyl indole to afford the substituted arene products. Compared to the highly negative potentials required for Shono's direct electroreductive protocol (<−2.5 V vs SCE),^[11]^ the photoelectrochemical protocol was successfully carried out at the reduction potential of the photocatalyst (−1.6 V vs SCE).^[206]^ This enables milder reaction conditions and indeed several functional groups, such as esters, amides, ethers, amines, unprotected alcohols and N‐heterocycles, were compatible with the developed protocol.

## 8 Concluding Remarks

**Figure 36:**
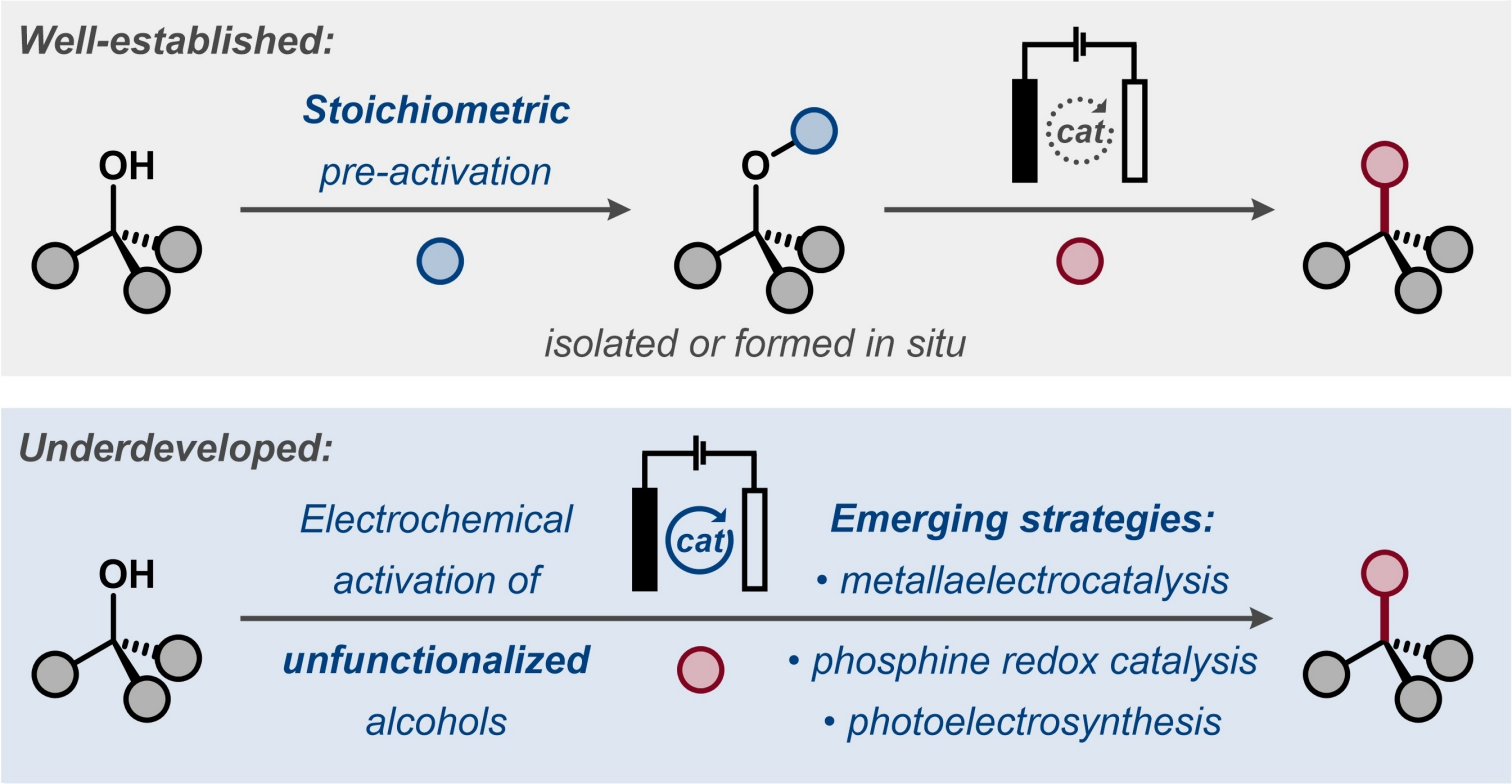
Established and emerging strategies for C−O bond activation.

The vast majority of currently existing electrosynthetic protocols for C−O bond activation and functionalization rely on in situ or ex situ stoichiometric derivatization of the parent alcohol. With such derivatization to form ethers, sulfonates, carbonyl compounds and phosphorous compounds, a plethora of direct and indirect methods have been developed to afford deoxygenated hydrocarbons as well as cross‐coupling products. The majority of these deoxygenative electrochemically driven protocols require a π‐system adjacent to the C−O bond to offer stabilization for high energy intermediates. In this context, transition metal catalysis was demonstrated to be a highly useful strategy for C−O bond cleavage for various alcohol derivatives at less reductive potentials compared to uncatalyzed electrosynthetic protocols. The use of phosphorous derivatives provides an alternative driving force for the C−O bond cleavage due to the formation of the thermodynamically stable P−O bond. Elegant recent examples that capitalize on this thermodynamic driving force were demonstrated for transition metal‐catalyzed cross‐coupling,^[197]^ as well as in a paired electrosynthetic protocol.^[179]^ Combined with contemporary progress in electrochemical phosphine oxide reduction,^[207]^ P‐based strategies are likely to open new avenues for fully catalytic deoxygenative functionalization of alcohols with electricity as the terminal reagent. In addition, the emerging field of photoelectrochemistry is highly promising and enables activation of the strong C−O bond under mild conditions, allowing high functional group tolerance.[^123^, ^205^, ^206^] While the available protocols for electrochemically driven C−O bond activation are often effective and selective, the need for pre‐functionalization of the alcohol is a drawback from both an atom‐ and step‐economy and a cost perspective. With only a few exceptions, unactivated alcohols remain a dormant precursor class for C‐centered radicals under electrochemical, photochemical and chemical conditions (Figure 36). Further development of strategies that capitalize on the combined effect of chemical affinity and redox mediating properties of transition metal catalysts for activation of non‐derivatized alcohols is yet to be realized. In this field, recent progress in non‐electrochemical deoxygenative transformation of alcohols using catalysts based on, for example, Cu,^[208]^ Ti,^[209]^ and Ni^[122]^ may serve as inspiration for future electrochemically driven protocols with improved atom economy for synthesis and late‐stage modification of complex organic molecules.

Electrosynthesis as a chemical production strategy aligns well with the contemporary electrification of the society to limit the dependence on fossil feedstocks and reduce CO_2_ emissions. With further progress in electrochemical C−O bond activation, renewable feedstocks rich in OH‐groups, such as lignocellulose, are expected to become available for new applications in material science and functional small molecule synthesis to further promote this transition.

## Conflict of interest

The authors declare no conflict of interest.

## Biographical Information

*Piret Villo received her Ph.D. in organic chemistry in 2013 after working with Dr. Lauri Vares at University of Tartu, and with Prof. Peter Somfai at KTH Royal Institute of Technology. She explored full synthesis of analogues to bioactive natural products, and as a separate project, asymmetric transfer hydrogenation of α‐amido‐β‐keto esters. Her postdoctoral studies on hypervalent iodine chemistry with Prof. Berit Olofsson at Stockholm University concentrated on transition metal‐free arylations with diaryliodonium salts. In 2019 she joined Asst. Prof. Helena Lundberg's group at KTH Royal Institute of Technology, where she studied Lewis acid catalyzed transformations, and is currently investigating electroreductive C−OH functionalization*.

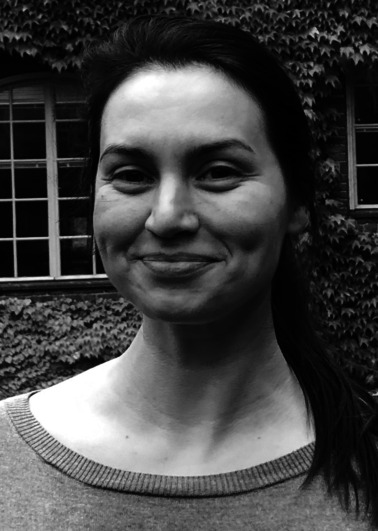

## Biographical Information

*Andrey Shatskiy completed a master's program in organic chemistry at Stockholm University in 2014 and subsequently joined the group of Prof. Björn Åkermark at Stockholm University as a Ph.D. student. His graduate studies were focused on the development and mechanistic studies of ruthenium‐based water‐oxidation catalysts. He received his Ph.D. degree in 2018 and then joined the Kärkäs group as a postdoctoral researcher at KTH Royal Institute of Technology, Stockholm. Currently, his research interests include photoredox catalysis, solar fuels, and organic electrosynthesis*.

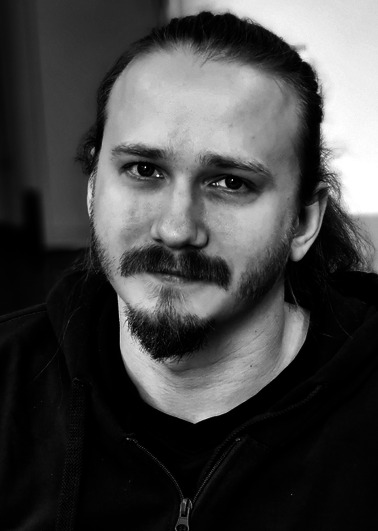

## Biographical Information

*Markus D. Kärkäs received his undergraduate degree from Stockholm University in 2008. In the same year he began his graduate studies under the direction of Prof. Björn Åkermark at Stockholm University. After receiving his Ph.D. degree in 2013, he joined Prof. Corey Stephenson's research group at the University of Michigan as a postdoctoral fellow. In 2016, he returned to the Department of Organic Chemistry at Stockholm University. In 2018, he joined the Department of Chemistry at KTH Royal Institute of Technology as an Assistant Professor and was promoted to Associate Professor in 2022. His research interests include photoredox catalysis, organic electrosynthesis, and transition metal catalysis. He was awarded a Bürgenstock fellowship in 2018 and is a recipient of the 2022 Thieme Chemistry Journals Award*.

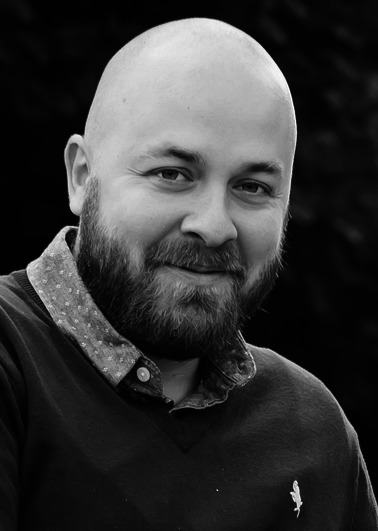

## Biographical Information

*Helena Lundberg received her Ph.D. degree in 2015 under the guidance of Prof. Hans Adolfsson at Stockholm University. After a year of postdoctoral research at Stockholm University with Prof. Fahmi Himo, she worked with Prof. Donna G. Blackmond and Prof. Phil S. Baran as postdoctoral fellow at Scripps Research in La Jolla, USA. In 2018, Helena joined KTH Royal Institute of Technology where she has been employed as Assistant Professor since 2021. Her research interests include catalysis and organic electrosynthesis, with a current focus on activation and functionalization of strong polarized σ‐bonds. In 2022, Helena was awarded a Bürgenstock fellowship and received a Future Research Leader grant from the Swedish Foundation of Strategic Research*.